# Crucial role of 4-deoxy-L-*erythro*-5-hexoseulose uronate reductase for alginate utilization revealed by adaptive evolution in engineered *Saccharomyces cerevisiae*

**DOI:** 10.1038/s41598-017-04481-3

**Published:** 2017-06-23

**Authors:** Fumiya Matsuoka, Makoto Hirayama, Takayuki Kashihara, Hideki Tanaka, Wataru Hashimoto, Kousaku Murata, Shigeyuki Kawai

**Affiliations:** 1 Laboratory of Basic and Applied Molecular Biotechnology, Division of Food Science and Biotechnology, Graduate School of Agriculture, Kyoto University, Uji, Kyoto 611-0011 Japan; 2 Faculty of Science and Engineering, Department of Life Science, Setsunan University, 17-8 Ikeda-Nakamachi, Neyagawa, Osaka 572-8508 Japan

## Abstract

In brown macroalgae, alginate and D-mannitol are promising carbohydrates for biorefinery. *Saccharomyces cerevisiae* is widely used as a microbial cell factory, but this budding yeast is unable to utilize either alginate or D-mannitol. Alginate can be depolymerized by both endo-type and exo-type alginate lyases, yielding a monouronate, 4-deoxy-L-*erythro*-5-hexoseulose uronate (DEH), a key intermediate in the metabolism of alginate. Here, we constructed engineered two *S. cerevisiae* strains that are able to utilize both DEH and D-mannitol on two different strain backgrounds, and we also improved their aerobic growth in a DEH liquid medium through adaptive evolution. In both evolved strains, one of the causal mutations was surprisingly identical, a c.50A > G mutation in the codon-optimized NAD(P)H-dependent DEH reductase gene, one of the 4 genes introduced to confer the capacity to utilize DEH. This mutation resulted in an E17G substitution at a loop structure near the coenzyme-binding site of this reductase, and enhanced the reductase activity and aerobic growth in both evolved strains. Thus, the crucial role for this reductase reaction in the metabolism of DEH in the engineered *S. cerevisiae* is demonstrated, and this finding provides significant information for synthetic construction of a *S. cerevisiae* strain as a platform for alginate utilization.

## Introduction

Brown macroalgae, containing the major carbohydrates alginate, D-mannitol, and laminarin, are promising carbon sources for production of biofuels and chemicals^1^. Alginate contains polyuronic acids, including the M-block (mannuronic acid residues), the G-block (guluronic acid residues), and the MG-block (alternating mannuronic acid and guluronic acid residues)^2^. Alginate comprises 13–20% of the fronds and 20–25% of the stipes in *Laminaria saccharina* ^3^ and 27–34% in *Sargassum horneri* ^4^. D-Mannitol (mannitol), a sugar alcohol corresponding to D-mannose, is oxidized to D-fructose by mannitol-2-dehydrogenase, generating NADH^5^. Mannitol comprises 9–23% of the fronds and 6–12% of the stipes in *L. saccharina* ^3^. Laminarin, a linear β-1,3-glucan (a polymer of glucose), comprises 1–21% of the fronds and 1–18% of the whole *L. saccharina* ^1^.

*Saccharomyces cerevisiae* is widely used as a microbial cell factory due to its genetic accessibility, robustness, high tolerance to both ethanol and inhibitory compounds under process conditions, and the considerable basic knowledge about this organism^6, 7^. Although *S. cerevisiae* is easily able to ferment glucose, a component of laminarin, this organism is unable to utilize either alginate or mannitol. Thus, it is challenging to use *S. cerevisiae* as a brown macroalgae-based biorefinery.

**Figure 1:**
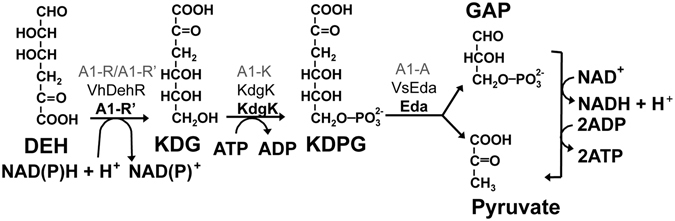
Metabolism of DEH. Enzymes in *Sphingomonas* sp. A1^9, 12, 19^ are in roman gray, those introduced in bioengineered *S. cerevisiae* by Enquist–Newman *et al*.^16^ are shown in black, and those introduced in bioengineered *S. cerevisiae* in this study are shown in bold black. Details are described in the text.

The metabolism of alginate is well characterized in a bacterium, *Sphingomonas* sp. A1, which is naturally able to utilize alginate (Fig. 1)^1, 8^. In *Sphingomonas* sp. A1, alginate is transported into the cell via a specific ABC-transporter, depolymerized by endo-type alginate lyases (A1-I, A1-II, A1-III) to oligoalginates, followed by degradation by exo-type alginate lyase, yielding an unsaturated uronate that is further non-enzymatically converted to 4-deoxy-L-*erythro*-5-hexoseulose uronate (DEH). NAD(P)H-dependent DEH reductases (A1-R and A1-R’) convert DEH to 2-keto-3-deoxy-D-gluconate (KDG), and KDG kinase (A1-K) phosphorylates KDG to 2-keto-3-deoxy-phosphogluconate (KDPG). KDPG aldolase (A1-A) cleaves KDPG to both pyruvate and glyceraldehyde-3-phosphate (GAP), and the latter is further metabolized to pyruvate^1, 8–11^. *Sphingomonas* sp. A1 was engineered to produce a maximum of 1.3 g of ethanol after 3 days in 100 mL medium containing 5 g sodium alginate with a feeding of 1 g of sodium alginate after 2 days^12^. This production was the first successful demonstration of the ability to generate valuable compounds from alginate. *Escherichia coli* is naturally unable to utilize alginate, although the genes for both KDG kinase and KDPG aldolase exist in *E. coli* genomic DNA as *kdgK* and *eda* ^13^. *E. coli* is able to utilize mannitol and was engineered to produce ethanol from alginate, producing 20 g/L of ethanol from 50 g/L of a sugar mixture (alginate, mannitol, and glucose at a ratio of 5:8:1) and 35–41 g/L of ethanol in 1 L of medium containing 130 g dry milled brown macroalgae (*L. japonica*, kombu)^13, 14^. Recently, it was reported that engineered *E. coli* produced L-lysine (43.3 mg/L) from oligoalginate depolymerized by alginate lyase AlyB^15^.

Based on the metabolism of alginate by *Sphingomonas* sp. A1, we began studies in 2012 to enable *S. cerevisiae* to utilize DEH to construct a yeast platform utilizing alginate. During our efforts, Enquist–Newman *et al*. succeeded for the first time in creating metabolically engineered *S. cerevisiae* that utilized DEH^16^. They identified a novel gene for the DEH transporter from an alginolytic eukaryote *Asteromyces cruciatus* and introduced the codon-optimized 4 genes required for metabolism of DEH into the genomic DNA of one *S. cerevisiae* strain. The 4 genes included the DEH transporter (Ac_DHT1), *Vibrio harvey* NAD(P)H-dependent DEH reductase (VhDehR), *E. coli* KdgK, and *Vibrio splendidus* KDPG aldolase (VsEda) (Fig. 1)^16^. Moreover, Enquist–Newman *et al*. identified the 2 genes needed for the metabolism of mannitol, including mannitol-2-dehydrogenase [YEL070W (Dsf1 or Man1) or YNR073C (Man2)] that oxidizes mannitol to D-fructose, and the putative MFS transporter [YEL069C (Hxt13) or YNR072W (Hxt17)]. These genes were identified using microarray analysis of the *S. cerevisiae* that had been “induced to grow efficiently in mannitol medium^16^” through an unknown mechanism. Enquist–Newman *et al*. introduced the 2 genes (*MAN2* and *HXT17*) into the genomic DNA of *S. cerevisiae* to artificially overexpress them. Thus, they succeeded in establishing a yeast strain that utilized mannitol, and they developed a yeast that is able to utilize both DEH and mannitol^16^. Recently, the mannitol-2-dehydrogenase activity of Man2 and Dsf1 and the mannitol transport activity of Hxt17 and Hxt13 were demonstrated^17^, and thus, we regard Hxt17/Hxt13 as a mannitol transporter. It should be emphasized that the aerobic growth efficiency of the engineered *S. cerevisiae* in a liquid medium containing DEH as a sole carbon source (the DEH medium) was improved through adaptive evolution; the initial doubling times of 16–64 h were reduced to 4–5 h after 100–150 generations^16^. Furthermore, a second adaptation improved the growth in a medium containing DEH plus mannitol under anaerobic conditions, resulting in the BAL3215 strain that produced 36.2 g/L of ethanol from 98 g/L of sugar (1:2 molar ratio of DEH:mannitol)^16^.

We recently reported that *S. cerevisiae* cells on a BY4742 background acquired the ability to utilize mannitol through spontaneous mutations in the genes for Tup1-Cyc8 corepressor, and the MK4416 strain carrying the *cyc8*Δ*1139–1164* allele exhibited the best ethanol productivity and salt-tolerance^18^. Thus, a substitution of the *cyc8*Δ*1139–1164* allele with native *CYC8* is an alternative method for enabling *S. cerevisiae* to utilize mannitol. At this time, it is not clear if this method or an artificial overexpression of *MAN2*/*HXT17* is more advantageous. Considering the potential importance of a brown macroalgae-based biorefinery using *S. cerevisiae*, construction of an engineered *S. cerevisiae* strain that utilizes DEH and mannitol at rates comparable to glucose, is indispensable. To achieve this, it is also crucial to understand the regulatory mechanisms in the metabolism of both DEH and mannitol as well as the mechanisms underlying the adaptations.

In this study, we initially evaluated which method was best to create a *S. cerevisiae* strain able to utilize mannitol. We successfully constructed engineered *S. cerevisiae* strains that were able to utilize both DEH and mannitol on two strain backgrounds (BY4742 and D452-2), and improved aerobic growth in DEH medium through adaptive evolution. We also identified one of the causal mutations that was unexpectedly the same in both evolved strains, a c.50A > G mutation in the gene for NAD(P)H-dependent DEH reductase (A1-R’). This mutation causes an E17G substitution at a loop structure that affected the coenzyme requirement^19^, and enhanced both the reductase activity and growth in DEH medium of both evolved strains.

## Results

### Enabling *S. cerevisiae* to utilize mannitol

*S. cerevisiae* wild type strains are unable to utilize mannitol, although this organism carries the gene (*DSF1*, *MAN2*) for mannitol-2-dehydrogenase^16–18, 20–23^. However, two reports using different methods have recently succeeded in getting *S. cerevisiae* to utilize mannitol: (i) an artificial overexpression of the genes for mannitol-2-dehydrogenase (Dsf1 or Man2) and for the mannitol transporter (Hxt13 or Hxt17)^16^, and (ii) replacement of the *cyc8*Δ*1139–1164* allele with native *CYC8* ^18^.

**Figure 2:**
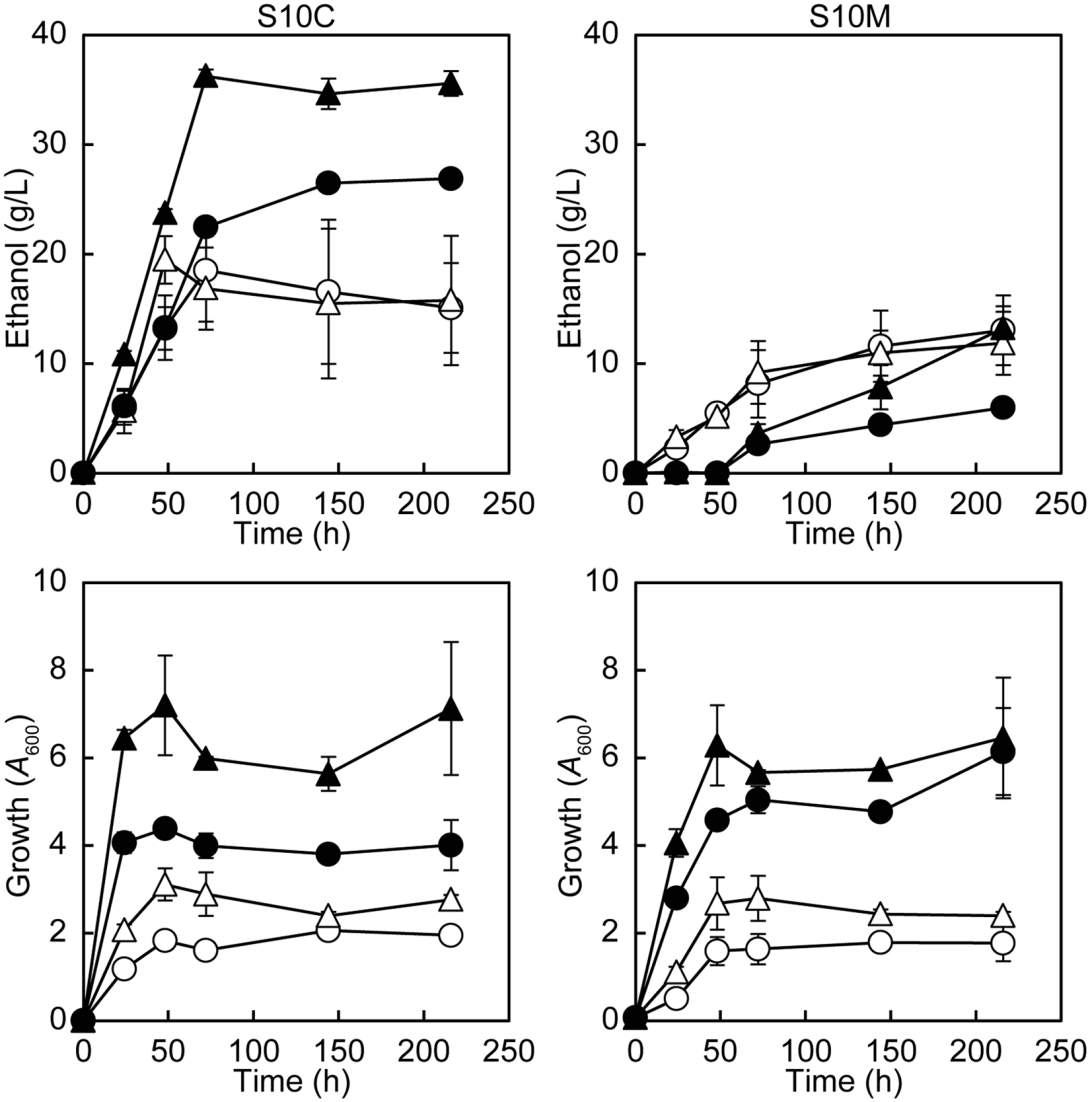
Ethanol productivity and growth of *S. cerevisiae* strains in both S10C and S10M media. Closed symbols; *S. cerevisiae* in which *DSF1* and *HXT17* were artificially overexpressed in BY4742 (MK5580, circle) and D452-2 (MK5583, triangle). Open symbols; *S. cerevisiae* BY4742 (MK4416, circle) and D452-2 (MK5502, triangle) that carry the *cyc8*Δ*1139–1164* allele. Cells were grown as described^18^. Briefly, cells from a glycerol stock were grown for approximately 3 days on YPGA solid medium, suspended in SDW, and inoculated into 50 ml of SC medium in a 100 ml Erlenmeyer flask at an *A*_600_ of 0.1. Cells were precultured for 24 h at 95 spm, washed once with SDW, resuspended in SDW, inoculated into 50 ml of S10C and into 50 ml of S10M in a 100 ml Erlenmeyer flask at an *A*_600_ of 0.1, and further cultivated at 95 spm as shown. Average and standard deviation (SD) are shown (n = 3).

To determine which method is better, we conferred the ability to utilize mannitol on two *S. cerevisiae* host strains (BY4742 and D452-2 strains) using the two methods described in Methods; (i) artificial overexpressions of the genes (*DSF1* and *HXT17*) that were integrated into the genomic DNA, yielding MK5580 (BY4742 background) and MK5583 (D452-2 background), and (ii) replacing the *cyc8*Δ*1139–1164* allele with *CYC8* in the genomic DNA of the D452-2 strain, resulting in MK5502. It should be noted that a spontaneous mutation occurred in *CYC8* in BY4742, creating *cyc8*Δ*1139–1164* and resulting in MK4416^18^. The D452-2 strain has been used for production of ethanol from various sugars, especially xylose^24–27^. We compared the ethanol productivity and growth of these 4 strains in S10C and in S10M media, which are the synthetic media containing 10% (w/v) glucose (S10C) and 10% (w/v) mannitol (S10M), respectively (Fig. 2). We found that the overexpression of *DSF1* and *HXT17* gave better results than replacing the *cyc8*Δ*1139–1164* allele with *CYC8*, except in the case of ethanol productivity in S10M. Moreover, we demonstrated that the activity of A1-R’ was not detected in the cell extract of the MK4416 strain (BY4742 *cyc8*Δ*1139–1164*) carrying pMK5090 (*yopt_A1-R’* in a plasmid pRS423), suggesting that the expression of *yopt_A1-R’* was severely impaired probably due to the *cyc8*Δ*1139–1164* allele. Based on these data, we decided to overexpress *DSF1* and *HXT17*.

### Genetic engineering in *S. cerevisiae* for DEH utilization

Enquist–Newman *et al*. reported that the 4 genes for *A. cruciatus* DEH transporter (Ac_DHT1), *V. harvey* NAD(P)H-dependent DEH reductase (VhDehR), *E. coli* KdgK, and *V. splendidus* KDPG aldolase (VsEda) are needed for *S. cerevisiae* to utilize DEH (Fig. 1)^16^. Enquist–Newman *et al*. optimized the codons of the 4 genes for *S. cerevisiae* and introduced each of the 4 genes flanked by a promoter and terminator into the genomic DNA of *S. cerevisiae* ^16^.

In order to generate *S. cerevisiae* with the ability to utilize DEH, we used the gene for the Ac_DHT1^16^, and we independently chose the genes for *Sphingomonas* sp. A1 NAD(P)H-dependent DEH reductase (A1-R’)^19^, *E. coli* KdgK^28^, and *E. coli* KDPG aldolase (Eda)^29^ (Fig. 1). *Yopt_kdgK*, *yopt_eda*, *yopt_A1-R’*, and *yopt_DHT1* are the genes with optimized codons for *S. cerevisiae* ^30^, and correspond to *E. coli kdgK* ^28^, *E. coli eda* ^29^, *Sphingomonas* sp. A1 *A1-R’* ^19^, and *A. cruciatus DHT1* ^16^. We synthesized *yopt_kdgK*, *yopt_eda*, and *yopt_A1-R’*, together with a promoter and terminator, and *yopt_DHT1* alone (Supplementary Figure S1). As described in the Supplementary Methods, *yopt_kdgK* (*TEF1p-yopt_kdgK-CYC1t*) and *yopt_eda* (*TEF1p-yopt_eda-CYC1t*) were introduced into a putative pseudogene (*YJL219w*) site in the genomic DNA of both MK5315 (BY4742 *ade2*Δ*0 trp1*Δ*63*) and D452-2, creating MK5524 and MK5517. Extracts of the resultant cells (MK5524 and MK5517) produced pyruvate (5.4 and 5.2 mM) from KDG, indicating that the introduced genes were functionally expressed. Then, *yopt_A1-R’* (*TEF1p-yopt_A1-R’-CYC1t*) and *yopt_DHT1* (*TDH3p-yopt*_*DHT1-TDH3t*) were integrated into a putative pseudogene (*YOL153C*) site in the genomic DNA of the above-mentioned MK5524 (BY4742 background) and MK5517 (D452-2 background), yielding MK5591 and MK5590. Extracts of the resultant cells (MK5591 and MK5590) showed DEH reductase activity (approximately 0.25 and 0.75 U/mg) and again produced pyruvate (0.36 and 0.10 mM) from DEH, demonstrating that the introduced *yopt_A1-R’*, *yopt_kdgK*, and *yopt_eda* were functionally expressed. Finally, *DSF1* (*TDH3p-DSF1-TDH3t*) and *HXT17* (*ADH1p-HXT17-ADH1t*) were introduced into a putative pseudogene (*YJL222w-A*) site in the genomic DNA of the above-mentioned MK5591 (BY4742 background) and also to the site around *TDH3t* in MK5590 (D452-2 background), resulting in MK5622 (BY_DEH+, BY4742 background) and MK5609 (D_DEH+, D452-2 background). Growth and ethanol production of the resultant BY_DEH+ and D_DEH+ strains in different media, including YP10C, YP10M, SC, and SM, were confirmed.

### Adaptive evolution of both BY_DEH+ and D_DEH+ in liquid DEH media

During the initial cultivation for 14 days, the MK5622 (BY_DEH+) and MK5609 (D_DEH+) strains reached an *A*_600_ of approximately 1.4 from a start of *A*_600_ 0.05 in a synthetic liquid medium containing DEH as a sole carbon source (initially, LC-LCA medium), while the parental strains (D452-2 and MK5315 [BY4742 *ade2*Δ *trp1*Δ*63*]) showed little growth reaching approximately *A*_600_ 0.2 from a start of *A*_600_ 0.05 after 14 days of cultivation, suggesting that the BY_DEH+ and D_DEH+ strains acquired a small ability to utilize DEH. The doubling times of the BY_DEH+ and D_DEH+ strains in the DEH (LC-LCA) liquid medium were calculated as 64 and 68 h, respectively.

This slow growth was in agreement with that previously reported by Enquist–Newman *et al*. where engineered *S. cerevisiae* strains exhibited slow growth in DEH medium, and the doubling time of the initial cultivation was 16–64 h^16^. Enquist–Newman *et al*. conducted adaptive evolution of these strains in a DEH medium under aerobic conditions in which the concentration of DEH was gradually increased to 17.6–35 g/L from an initial concentration of 8.8 g/L, and the doubling time was reduced to 4–5 h after 100–150 generations^16^. Thus, we also decided to conduct adaptive evolution of the BY_DEH+ and D_DEH+ strains in the liquid DEH medium aerobically as described in Methods. The DEH medium was finally optimized to the HC-LN medium. In the HC-LN medium, the initial doubling times of the BY_DEH+ and D_DEH+ strains were determined to be 25 and 36 h, respectively (Table 1). The BY_DEH+ and D_DEH+ strains successfully evolved to MK5719 (BY_DEH++) and MK5717 (D_DEH++), and the doubling times after 160 and 141 generations were improved to 10 and 11 h, respectively (Table 1).

**Table 1:** Adaptive evolution in DEH medium ^a^.

Strain		Background	Doubling time (h)	μ (h^−1^)	Generation	Time of subculture
MK5622	BY_DEH+	BY4742	25	0.028	—	—
MK5719	BY_DEH++	BY4742	10	0.069	160	30
MK5609	D_DEH+	D452-2	36	0.019	—	—
MK5717	D_DEH++	D452-2	11	0.062	141	28

^a^The MK5622 (BY_DEH+) and MK5609 (D_DEH+) strains evolved to the MK5719 (BY_DEH++) and to the MK5717 (D_DEH++) strains, respectively, in DEH liquid medium aerobically. All doubling times were determined using optimized HC-LN liquid medium.

### The DEH+ and DEH++ strains utilize DEH as a non-fermentable carbon source

**Figure 3:**
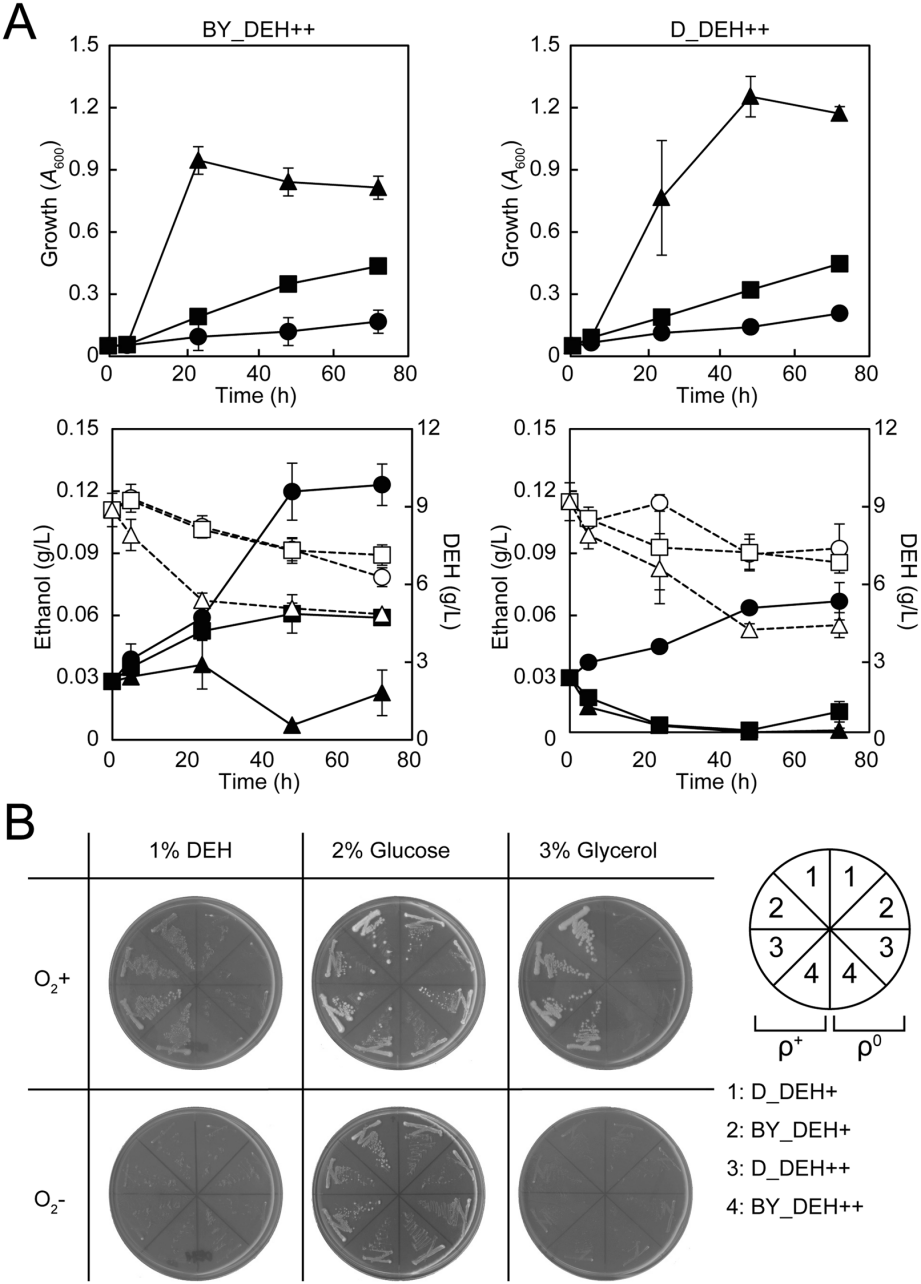
DEH+ and DEH++ strains require mitochondria and oxygen to utilize DEH. (**A**) Aeration improved growth, but repressed ethanol production. Each strain was cultured in 1.0 mL of liquid DEH medium (HC-LN medium) in a test tube at 0 spm (circles), 95 spm (squares), and 145 spm (triangles) at 30 °C. The growth and concentration of ethanol are shown with closed symbols and those of DEH are shown with open symbols with dashed lines. The average and SD are shown (n = 3). (**B**) DEH+ and DEH++ strains utilize DEH as a non-fermentable carbon source. Strains were grown either aerobically (+O^2^) or anaerobically (− O^2^) for 15 days. Either DEH or glucose was included in the synthetic medium, while glycerol was in the YP medium.

Both BY_DEH++ and D_DEH++ strains were grown in 1.0 mL DEH medium (HC-LN medium) in test tubes either without shaking (0 strokes per min; spm) or with shaking at 95 and 145 spm. In the cases of both BY_DEH++ and D_DEH++ strains, aeration improved growth and consumption of DEH, but repressed ethanol production (Fig. 3A), suggesting that metabolism of DEH requires respiration with a functional mitochondria. Therefore, we created the ρ° mutants of both DEH+ and DEH++ strains on both backgrounds, in which the mitochondrial respiration was lost due to a lack of mitochondrial genomic DNA^31^. We then tested the growth of the ρ° mutants and the ρ^+^ parents on solid media containing either DEH, glucose, or glycerol under aerobic or anaerobic condition (Fig. 3B). Under anaerobic conditions, all of the strains failed to grow on either DEH or glycerol but did grow on glucose (fermentable carbon source). Under aerobic conditions, the ρ^0^ mutants were unable to grow but the ρ^+^ parents were able to grow on either DEH or glycerol. Both ρ^0^ mutants also showed no aerobic growth in liquid DEH medium (data not shown). These data unequivocally indicated that mitochondria and oxygen were essential to metabolize DEH. Both DEH+ and DEH++ strains utilize DEH as a non-fermentable carbon source. We also checked the growth of these strains in mannitol-containing medium and demonstrated that both DEH+ and DEH++ strains also utilize mannitol as a non-fermentable carbon source, in agreement with our previous results^18^.

### Mechanism underlying adaptive evolution

We initially focused on mitochondrial function to determine how DEH+ strains have evolved to DEH++ strains on both backgrounds. We hypothesized that mitochondrial respiratory functions were improved in both DEH++ strains, since utilization of DEH requires mitochondrial function (Fig. 3). Oxygen consumption rates for both DEH++ strains and DEH+ strains that were grown in SE liquid medium were measured. However, we observed no difference in oxygen consumption rates between the DEH+ strains and DEH++ strains (Supplementary Fig. S2A). We were unable to measure the oxygen consumption rates of the DEH+ strains in DEH medium due to their slow growth.

**Figure 4:**
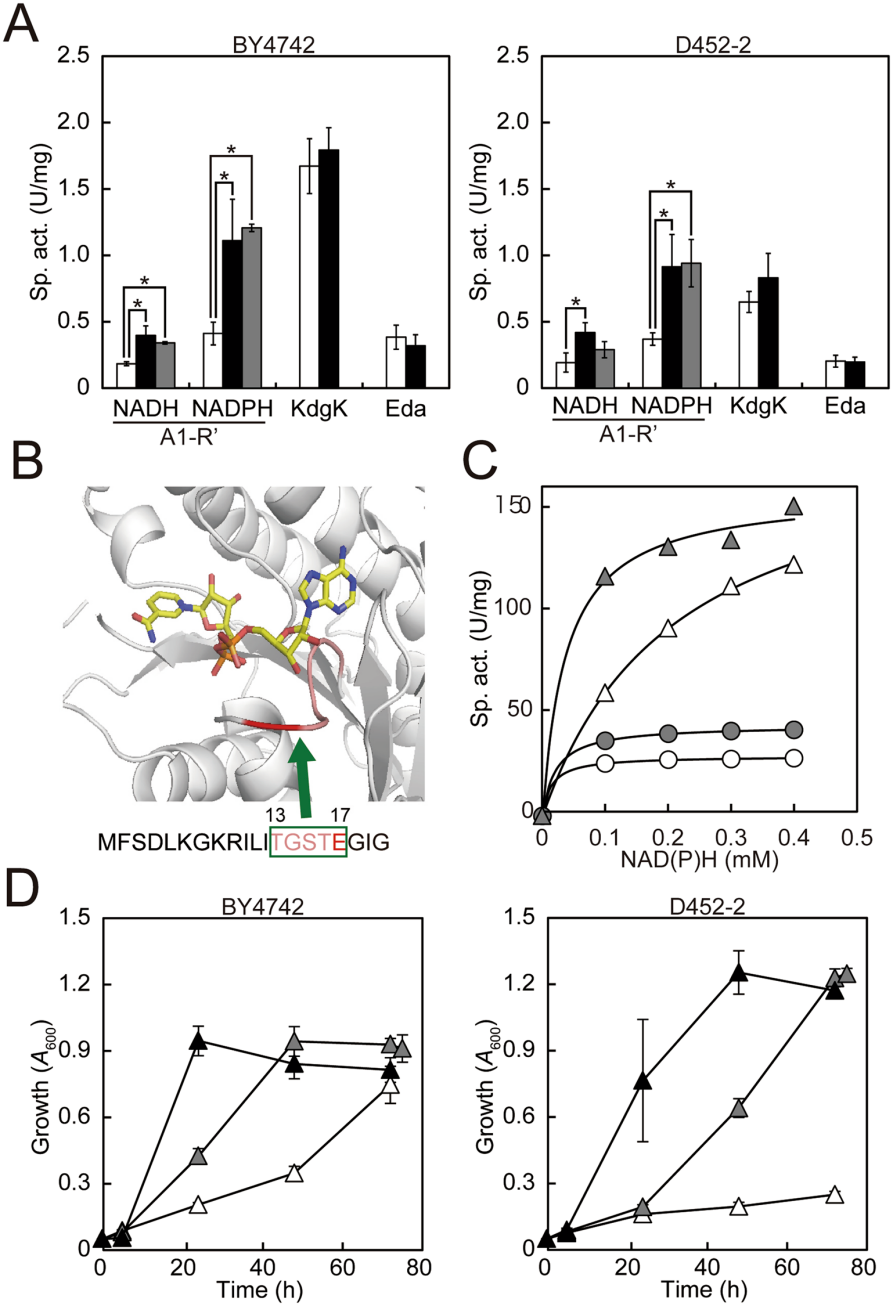
The c.50A > G mutation in *yopt_A1-R’* was the causal mutation for enhanced DEH reductase activity and for aerobic growth in DEH medium. (**A**) Enzymatic activities. Cell extracts were made from DEH+ (white bar), DEH++ (black bar), and DEH+_E17G (gray bar) strains on either the BY4742 or D452-2 background. Activities were assayed as in Methods. *p < 0.05. (**B**) Crystal structure of A1-R’ complexed with NAD^+ ^^19^. The loop structure is shown in pink, and Glu-17 is marked in red. The N-terminal sequence of the primary structure of A1-R’ is shown, in which the loop structure (^13^TGSTE^17^) is boxed in green. The figure of the structure was prepared with the PyMOL program using the coordinates of the A1-R’/NAD^+^ complex (PDB: 4TKM)^19^. (**C**) NADH (circles)- and NADPH (triangles)-dependent specific activities of the purified rA1-R’ (open symbols) and rA1-R’_E17G (gray symbols). (**D**) Growth of the DEH+ (open triangles), DEH++ (closed triangles), and DEH+_E17G (gray triangles) strains on either the BY4742 or D452-2 background. Yeast cells were grown as in Fig. 3A at 145 spm. (**A,D**) Average and SD are shown (n = 3).

Next, we focused on the 4 introduced genes: *yopt_kdgK*, *yopt_eda*, *yopt_A1-R’*, and *yopt_DHT1*. We hypothesized that the high up-regulation of transcription of the 4 genes might affect adaptive evolution. However, transcription of the 4 genes was not highly up-regulated in both DEH++ strains grown in DEH medium. In fact, the transcription of *yopt_kdgK* in both DEH++ strains was slightly down-regulated, and the transcription of the other genes was only slightly different (Supplementary Fig. S2B). We also measured the catalytic activities of A1-R’, KdgK, and Eda using cell extracts of the 4 strains grown in DEH medium and found that only the activities of A1-R’ (DEH reductase) were increased in both DEH++ strains (Fig. 4A, Fig. S2C). The nucleotide sequences of *yopt_kdgK*, *yopt_eda*, and *yopt_DHT1* in both DEH++ strains were normal, but a c.50A > G mutation in *yopt_A1-R’* in both DEH++ strains was detected. The *yopt_A1-R’* in both DEH+ strains was confirmed again to lack this mutation. The c.50A > G mutation in *yopt_A1-R’* causes a p.Glu17Gly (E17G) substitution in A1-R’ (Fig. 4B).

The c.50A > G mutation was artificially introduced into *yopt_A1-R’*, creating *yopt_A1-R’*(c.50A > G), to verify whether the E17G substitution enhances DEH reductase activity. *A1-R’* and *yopt_A1-R’*(c.50A > G) were overexpressed in *E. coli* as recombinant proteins, purified, and the kinetic values of the purified recombinant proteins (rA1-R’ and rA1-R’_E17G) were determined (Table 2). rA1-R’_E17G is the rA1-R’ in which Glu-17 is substituted with a Gly residue. The saturation curves of the purified enzymes clearly showed that the E17G substitution enhanced the reductase activity (Fig. 4C). The kinetic values showed that the E17G substitution slightly increased the *K*_m_ and *k*_cat_ for NADH and the *k*_cat_ for DEH, but largely reduced the *K*_m_ for NADPH and increased the *K*_m_ for DEH (Table 2). Furthermore, the *yopt_A1-R’*(c.50A > G) was replaced with *yopt_A1-R’* in the genomic DNA of both DEH+ strains as described in Methods, yielding MK5909 (BY_DEH+ _E17G) and MK5906 (D_DEH+ _E17G), respectively. The NAD(P)H-dependent DEH reductase activities of cell extracts from both DEH+ _E17G strains were higher than those from both DEH+ strains (Fig. 4A, gray bars vs white bars).

**Table 2:** Kinetic parameters of the purified rA1-R’ and rA1-R’_E17G.

	rA1-R’			rA1-R’_E17G		
	*k*_cat_	*K*_m_	*k*_cat_ *K*_m_^−1^	*k*_cat_	*K*_m_	*k*_cat_ *K*_m_^−1^
	s^−1^	μM	s^−1^ μM^−1^	s^−1^	μM	s^−1^ μM^−1^
DEH^a^	53.5 ± 0.3	794 ± 30	0.0675	118 ± 10	2,354 ± 652	0.0501
NADH	53.1 ± 0.3	13.4 ± 1.2	3.97	80.1 ± 0.1	20.7 ± 0.2	3.86
NADPH	344 ± 7	214 ± 10	1.61	287 ± 14	37.9 ± 11.0	7.58

^a^NADH was used for the assay.

We next investigated whether the enhanced DEH reductase activity due to the E17G substitution improved aerobic growth in the DEH liquid medium. We compared the growth of both DEH+ _E17G strains with the growth of both DEH+ strains and both DEH++ strains (Fig. 4D). In the DEH liquid medium, both DEH+ _E17G strains exhibited obvious improved growth over that of both DEH+ strains, and finally reached the same growth as both DEH++ strains at day 3 (Fig. 4D). Delayed growth at either day 1 or 2 of both DEH+_E17G strains was observed compared with that of both DEH++ strains, thus demonstrating that the enhanced DEH reductase activity due to the c.50A > G mutation in *yopt_A1-R’* was one of the crucial events responsible for adaptive evolution. We propose that the delayed growth of both DEH+_E17G strains at days 1 or 2 could be attributed to other events rather than this causal mutation.

## Discussion

This is the second report that succeeded in creating a synthetically engineered *S. cerevisiae* strain capable of utilizing both DEH and mannitol. The first success was reported by Enquist-Newman *et al*. who integrated the 4 genes needed for DEH metabolism and the 2 genes needed for mannitol metabolism into the genomic DNA of one *S. cerevisiae* strain^16^. In this study, we also introduced 4 genes for the metabolism of DEH and the 2 genes for the metabolism of mannitol into the genomic DNAs of two *S. cerevisiae* strains (BY4742 and D452–2). It should also be noted that the 2 genes for DEH metabolism (*yopt_A1-R’* and *yopt_eda*) were different from those previously reported^16^ (Fig. 1). Both Enquist-Newman *et al*. and our group conducted adaptive evolution in DEH medium and succeeded in obtaining evolved strains with enhanced aerobic growth in DEH medium. Here, we found that DEH and mannitol are utilized as non-fermentable carbon sources by our engineered *S. cerevisiae* and identified, for the first time, one casual mutation, the c50.A > G mutation in *yopt-A1-R’*, that enhances the growth in DEH medium after adaptive evolution.

It was surprising that the two DEH+ strains had the same causal mutation in the same gene, *yopt_A1-R’*, during independent adaptive evolution studies. This mutation causes an E17G substitution in A1-R’ and enhanced both NADH- and NADPH-dependent DEH reductase activities (Fig. 4A,C, Supplementary Fig. S2C, Table 2). Notably, based on the crystal structure, Glu-17 in A1-R’ locates at the crucial loop structure of A1-R’ (Fig. 4B)^19^. The loop structure locates near the ribose moiety in nicotinamide ribose in the bound NAD^+^ molecule (Fig. 4B) and is one of the two determinants for coenzyme specificity^19^. Our data here again emphasize the prominent role of the loop structure for the catalytic activity of A1-R’. Moreover, this study demonstrates the crucial role of NAD(P)H-dependent DEH reductase reactions for DEH metabolism. The data are important for conferring the ability to utilize DEH on other *S. cerevisiae* strains. However, the substitution did not fully explain the evolution. Both DEH+_E17G strains exhibited delayed growth at day 1 (BY_ DEH+_E17G) and at days 1 and 2 (D_ DEH+_E17G) (Fig. 4D). A full understanding of the mechanism for the adaptive evolution has not been established.

Moreover, Enquist-Newman *et al*. further conducted the second adaptive evolution under anaerobic conditions involving both mannitol and DEH^16^. These adaptive evolutions in DEH plus mannitol medium are similar to the evolution of engineered *S. cerevisiae* strains capable of utilizing xylose, one of the major sugars in plant biomass: from parental strain 22–3 to strain 127 through aerobic batch evolution with 0.1% glucose plus 2% xylose, and finally to strain 128 through anaerobic batch evolution with 0.1% glucose plus 2% xylose^32^. Furthermore, a surprising connection between Fe-S cluster biogenesis and signaling that facilitates aerobic respiration and anaerobic fermentation of xylose has been suggested in these evolutions^33^. Complete clarification of the mechanism underlying the adaptive evolution under anaerobic conditions in mannitol plus DEH as well as the regulatory mechanism in the metabolism of DEH and mannitol will be the next challenge.

## Methods

### Plasmids, strains, and primers

The genes, *yopt_kdgK*, *yopt_eda*, *yopt_A1-R’*, *yopt_DHT1*, and *yopt_A1-I* that correspond to *E. coli kdgK* ^28^, *E. coli eda* ^29^, *Sphingomonas* sp. A1 *A1-R’* ^19^, *A. cruciatus DHT1* ^16^ (Fig. 1), and *Sphingomonas* sp. A1 *A1-I* ^34^, with the codons optimized for *S. cerevisiae* ^30^, were synthesized (Supplementary Fig. S1). The plasmids, strains, and primers used in this study are listed in Supplementary Tables S1, S2 and S3. Details on the construction of the plasmids and the strains are described in Supplementary Methods.

### Preparation of DEH

Recombinant Atu3025 was expressed and purified as described^35^. Recombinant A1-I^34^ was expressed in MK5632 and purified as described^35^, but at 16 °C, not at 30 °C. We added 0.5 mL of the purified A1-I (1.46 mg/mL, 7.81 U/mg) and 1 mL of the purified Atu3025 (2.15 mg/mL, 8.57 U/mg) to the sodium alginate [100 mL, 1.0% (w/v) in water]. The mixture was incubated at 30 °C, 100 spm, for 18 h. The resultant solution was concentrated to approximately 10 mL with a freeze dryer. High molecular weight compounds, such as proteins, were removed from the concentrated solution with a Centriprep-10K centrifugal filter (Merck Millipore, Massachusetts, USA) (NMWL 10,000) using centrifugation at 1,600 g at 4 °C for 45–60 min. The resultant solution was filtered through a cellulose acetate filter (pore size, 0.2 μm). The concentration of DEH was determined as described with the molecular coefficient of DEH at *A*_548_ (ε = 58,000 M^−1^cm^−1^)^35^.

### Medium and growth conditions

Standard yeast media were used^18, 36^. YPD, YPM, and YPG media contained YP (1% yeast extract, 2% tryptone, pH 5.6) with 2% (w/v) glucose, 2% (w/v) mannitol, and 3% (v/v) glycerol, respectively. YPDA and YPGA were the YPD and YPG media containing 15 mg/L of adenine. YP10C and YP10M consisted of YP with 10% (w/v) glucose and 10% (w/v) mannitol. SC, SM, and SE were synthetic media consisting of 0.67% (w/v) yeast nitrogen base without amino acids (YNB w/o aa) (BD) and complete amino acids/nucleosides (Clontech) with either 2% (w/v) glucose, 2% (w/v) mannitol, or 2% (w/v) ethanol, respectively. S10C and S10M were SC and SM media containing either 10% (w/v) glucose or 10% (w/v) mannitol. LC-LCA medium was the initial DEH medium, and was the screening medium used^16^, but it contained 0.5 g/L MgSO_4_ instead of 0.5 mg/L MgCl_2_, and also included 0.5% w/v DEH, 20 mM MES (pH 5.6), 0.69 g/L -Leu DO Supplement (Clontech, Mountain View, CA), and an amino acid mixture (0.11 g/L Ade, 0.91 g/L Asp, 1.14 g/L Leu, 0.57 g/L Ile, 1.6 g/L Val, and 0.23 g/L Met). HC-LN medium was the optimized DEH medium consisting of 0.17% YNB, without amino acids and without ammonium sulfate, 1.0% (w/v) DEH, 20 mM MES (pH 5.6), 0.69 g/L -Leu DO Supplement, 5 mM (0.66 g/L) Asn, and 200 mg/L geneticin. HC-LN medium was used as a DEH medium, unless otherwise stated. For the DEH+_E17G strains, HC-LN medium without geneticin was used. Medium was solidified with 2% (w/v) agar. Liquid media were used, unless otherwise specified. Cells were stored at −80 °C in the presence of 17% (v/v) glycerol. Yeast cells were cultivated at 30 °C. A PersonalLt-10F (Taitec, Japan) was used to shake the cultures. Anaerobic growth was conducted using an AnaeroPack (Mitsubishi Gas Chemical).

### Adaptation of both MK5609 (D_DEH+) and MK5622 (BY_DEH+) to the DEH medium

MK5609 and MK5622 strains from the glycerol stocks were regrown on YPDA solid medium, inoculated into 1.5 mL LC-LCA medium, and further cultivated at 30 °C. These strains were cultivated for 2 weeks for the 1–34 and 1–36 generations and for 2–3 days for the 35–141 and 37–160 generations of MK5609 and MK5622, respectively. The initial *A*_600_ was 0.05 for both the 1–30 and 1–34 generations. During the 31–141 and 35–160 generations, the cultures were diluted by 1/10– to 1/30-fold in the same fresh medium. LC-LCA medium was used for the 1–11 and 1–12 generations. After 12 and 13 generations of both MK5609 and MK5622, respectively, 200 mg/L geneticin was added to the medium. DEH was included at 0.5% (w/v) for the 1–67 and 1–75 generations and at 1.0% (w/v) for the 68–141 and 76–160 generations. The other amino acid mixtures were replaced with 0.66 g/L Asn during the 68–141 and 76–160 generations. Throughout the adaptive evolutions, the cells were cultured at 145 spm. The medium was finally optimized to HC-LN medium. Cultures of MK5609 and MK5622 strains that had passed 141 and 160 generations (28 and 30 times of subculture) were streaked on YPGA solid medium to obtain single colonies. The growth of each of the single colonies in HC-LN containing DEH as a sole carbon source was examined, and the strains exhibiting the best growth were selected as MK5719 (BY_DEH++) and MK5717 (D_DEH++), respectively.

### Assays

The concentration of ethanol was assayed, and cell extracts of *S. cerevisiae* were prepared as described^18^. The protein concentration was determined with the Bradford reagent (Sigma-Aldrich)^37^. Kinetic parameters were determined by fitting the data to the appropriate Michaelis-Menten equation using KaleidaGraph software (Synergy Software).

DEH reductase (A1-R’) activity was continuously measured by monitoring the decrease in *A*_340_ at 30 °C as described^19^ in a reaction mixture (500 μL; 0.15 mM [for assay of DEH reductase activity in yeast cell extracts] or in either 0.2 mM [for purified enzymes] NADPH or NADH, 4.3 mM DEH, and enzyme preparations (either yeast cell extracts [3.0–50 μg], purified rA1-R’ [68–203 ng], or purified rA1-R’_E17G [33–98 ng]). KdgK activity was measured with the stop method. A reaction mixture (35 μL; 50 mM potassium phosphate, pH 7.0, containing 10 mM ATP, 10 mM MgSO_4_, 10 mM KDG [Sigma-Aldrich], a partially purified Eda [3.2 μg, 60 U], and *S. cerevisiae* cell extracts [3.5 μg]) was incubated at 30 °C for various times (2.5 – 5.0 min), boiled for 5 min, and centrifuged at 20,000 g for 10 min. The amount of pyruvate produced in the supernate was determined as described^38^. Eda was over-expressed in ASKA (−) JW1839 (eda) as described^39^, but required a 5.5 h induction with 0.1 mM IPTG at 37 °C, and was partially purified by TALON affinity chromatography as described^40^. For the assay of Eda activity, pyruvate production was continuously monitored in a reaction mixture (500 μL; 300 mM triethanolamine HCl/5 mM EDTA, pH 7.6^38^, 0.15 mM NADH, 0.2 mM KDPG [Sigma-Aldrich], 4.2 U L-lactate dehydrogenase from rabbit muscle, and *S. cerevisiae* cell extracts [5.0 – 30 μg]) at 30 °C. One unit of each enzyme (A1-R’, KdgK, and Eda) was defined as the amount of enzyme required to generate 1 μmol of product per min.

For the assay of pyruvate production from KDG with *S. cerevisiae* cell extracts, the reaction mixture (70 μL) consisted of 10 mM ATP, 10 mM MgSO_4_, 50 mM potassium phosphate, pH 7.0, *S. cerevisiae* cell extracts (0.25 – 0.50 mg proteins/mL), and 10 mM KDG. In the case of pyruvate production from DEH, the reaction mixture was the same but included 10 mM DEH plus 10 mM NADPH instead of 10 mM KDG. The reaction mixtures were incubated at 30 °C for 5 min, boiled for 5 min, and centrifuged at 20,000 g at 4 °C for 10 min. The pyruvate concentration in the resultant supernate was determined with an F kit Pyruvate (Nacalai).

### Quantitative PCR

*S. cerevisiae* strains stored at −80 °C were cultured on YPGA solid medium, suspended in SDW, and inoculated in 10 mL HC-LN medium to reach an *A*_600_ of 0.05 in a 50 mL Erlenmeyer flask, and cultivated at 145 spm at 30 °C to reach an *A*_600_ of 0.53 (MK5609 [D_DEH+], 3d-cultivation), *A*_600_ 1.01 (MK5622 [BY_DEH+], 3d-cultivation), *A*_600_ 1.65 (MK5717 [D_DEH++], 2 d-cultivation), and *A*_600_ 1.05 (MK5719 [BY_DEH++], 2 d-cultivation). RNA was extracted and purified, and quantitative PCR (qPCR) was conducted as described^41^. Primers 32–41 were used (Supplementary Table S3).

### Nucleotide sequences of the introduced 4 genes

To sequence the nucleotides of the introduced 4 genes in the BY_DEH+, BY_DEH++, D_DEH+, and D_DEH++ strains, the genes were amplified with genomic PCR using KOD-Plus Neo (Toyobo, Japan) and each genomic DNA as a template. The primers were 23 and 25 for *yopt_A1-R’*, primers 23 and 18 for *yopt_kdgK*, primers 22 and 21 for *yopt_eda*, and primers 24 and 21 for *yopt_DHT1*. For sequencing, the primers were 48 and 49 for *yopt_A1-R’* and *yopt_kdgK*, primer 48 for *yopt_eda*, and primers 28 and 30 for *yopt_DHT1*.

### Purification of both rA1-R’ and rA1-R’_E17G

Recombinant A1-R’ (rA1-R’) and A1-R’_E17G (rA1-R’_E17G) were expressed in both MK4700 and MK5923 and were purified as reported previously^19^.

## Electronic supplementary material

Supplementary information

## References

[1] Kawai, S. & Murata, K. Biofuel production based on carbohydrates from both brown and red macroalgae: recent developments in key biotechnologies. *Int. J. Mol. Sci*. **17**, doi:10.3390/ijms17020145 (2016). 10.3390/ijms17020145 PMC4783879 26861307

[2] Larsen B, Salem DMSA, Sallam MAE, Mishrikey MM, Beltagy AI (2003). Characterization of the alginates from algae harvested at the Egyptian Red Sea coast. Carbohydr. Res..

[3] Black WAP (1950). The seasonal variation in weight and chemical composition of the common British Laminariaceae. J. Mar. Biol. Assoc. U.K..

[4] Kimura T (2007). The seasonal variation in polysaccharide content of brown alga akamoku *Sargassum horneri* collected off Oshima Island (Fukuoka Prefecture). Nippon Suisan Gakkaishi.

[5] Horn SJ, Aasen IM, Østgaard K (2000). Production of ethanol from mannitol by *Zymobacter palmae*. J. Ind. Microbiol. Biotechnol..

[6] Hughes, S. R. & Qureshi, N. in Biomass to biofuels: strategies for global industries. (eds A. Vertès, N. Qureshi, H. Yukawa & H.P. Blaschek) 55–69 (Wiley, 2010).

[7] Nielsen J, Larsson C, van Maris A, Pronk J (2013). Metabolic engineering of yeast for production of fuels and chemicals. Curr. Opin. Biotechnol..

[8] Murata K, Kawai S, Mikami B, Hashimoto W (2008). Superchannel of bacteria: biological significance and new horizons. Biosci. Biotechnol. Biochem..

[9] Takase R, Ochiai A, Mikami B, Hashimoto W, Murata K (2010). Molecular identification of unsaturated uronate reductase prerequisite for alginate metabolism in *Sphingomonas* sp. A1. Biochim. Biophys. Acta.

[10] Preiss J, Ashwell G (1962). Alginic acid metabolism in bacteria. I. Enzymatic formation of unsaturated oligosac-charides and 4-deoxy-L-erythro-5-hexoseulose uronic acid. J. Biol. Chem..

[11] Preiss J, Ashwell G (1962). Alginic acid metabolism in bacteria. II. The enzymatic reduction of 4-deoxy-L-erythro-5-hexoseulose uronic acid to 2-keto-3-deoxy-D-gluconic acid. J. Biol. Chem..

[12] Takeda H, Yoneyama F, Kawai S, Hashimoto W, Murata K (2011). Bioethanol production from marine biomass alginate by genetically engineered bacteria. Energy. Environ. Sci..

[13] Wargacki AJ (2012). engineered microbial platform for direct biofuel production from brown macroalgae. Science.

[14] Santos, C. N., Regitsky, D. D. & Yoshikuni, Y. Implementation of stable and complex biological systems through recombinase-assisted genome engineering. *Nat. Commun*. **4**, 2503 doi:2510.1038/ncomms3503 (2013). 10.1038/ncomms3503 24056574

[15] Doi, H. *et al*. Identification of enzymes responsible for extracellular alginate depolymerization and alginate metabolism in *Vibrio algivorus*. *Appl. Microbiol. Biotechnol*. (2016). 10.1007/s00253-016-8021-7 PMC5266763 27915375

[16] Enquist-Newman M (2014). Efficient ethanol production from brown macroalgae sugars by a synthetic yeast platform. Nature.

[17] Jordan, P., Choe, J. Y., Boles, E. & Oreb, M. Hxt13, Hxt15, Hxt16 and Hxt17 from *Saccharomyces cerevisiae* represent a novel type of polyol transporters. *Sci Rep* **6**, 23502 doi:23510.21038/srep23502 (2016). 10.1038/srep23502 PMC4800717 26996892

[18] Chujo M, Yoshida S, Ota A, Murata K, Kawai S (2015). Acquisition of the ability To assimilate mannitol by *Saccharomyces cerevisiae* through dysfunction of the general corepressor Tup1-Cyc8. Appl. Environ. Microbiol..

[19] Takase R, Mikami B, Kawai S, Murata K, Hashimoto W (2014). Structure-based conversion of the coenzyme requirement of a short-chain dehydrogenase/reductase involved in bacterial alginate metabolism. J. Biol. Chem..

[20] Barnett JA (1968). The catabolism of acyclic polyols by yeasts. J. Gen. Microbiol..

[21] Quain DE, Boulton CA (1987). Growth and metabolism of mannitol by strains of *Saccharomyces cerevisiae*. J. Gen. Microbiol..

[22] Ota A, Kawai S, Oda H, Iohara K, Murata K (2013). Production of ethanol from mannitol by the yeast strain *Saccharomyces paradoxus* NBRC 0259. J. Biosci. Bioeng..

[23] Perfect JR (1996). Identification of a *Cryptococcus neoformans* gene that directs expression of the cryptic *Saccharomyces cerevisiae* mannitol dehydrogenase gene. J. Bacteriol..

[24] Lee SH, Kodaki T, Park YC, Seo JH (2012). Effects of NADH-preferring xylose reductase expression on ethanol production from xylose in xylose-metabolizing recombinant *Saccharomyces cerevisiae*. J. Biotechnol..

[25] Watanabe S (2007). Ethanol production from xylose by recombinant *Saccharomyces cerevisiae* expressing protein-engineered NADH-preferring xylose reductase from *Pichia stipitis*. Microbiology.

[26] Khattab SMR, Kodaki T (2014). Efficient bioethanol production by overexpression of endogenous *Saccharomyces cerevisiae* xylulokinase and NADPH-dependent aldose reductase with mutated strictly NADP^+^-dependent *Pichia stipitis* xylitol dehydrogenase. Process Biochem..

[27] Ha SJ (2011). Engineered *Saccharomyces cerevisiae* capable of simultaneous cellobiose and xylose fermentation. Proc. Natl. Acad. Sci. USA.

[28] Cynkin MA, Ashwell G (1960). Uronic acid metabolism in bacteria. 4. Purification and properties of 2-keto-3-deoxy-D-gluconokinase in *Escherichia coli*. J. Biol. Chem..

[29] Egan SE (1992). Molecular characterization of the Entner-Doudoroff pathway in *Escherichia coli*: sequence analysis and localization of promoters for the *edd-eda* operon. J. Bacteriol..

[30] Bennetzen JL, Hall BD (1982). Codon selection in yeast. J. Biol. Chem..

[31] Fox TD (1991). Analysis and manipulation of yeast mitochondrial genes. Methods Enzymol.

[32] Parreiras, L. S. *et al*. Engineering and two-stage evolution of a lignocellulosic hydrolysate-tolerant *Saccharomyces cerevisiae* strain for anaerobic fermentation of xylose from AFEX pretreated corn stover. *Plos One* **9** (2014). 10.1371/journal.pone.0107499 PMC4164640 25222864

[33] Sato TK (2016). Directed evolution reveals unexpected epistatic interactions that alter metabolic regulation and enable anaerobic xylose use by *Saccharomyces cerevisiae*. PLoS Genet.

[34] Yoon HJ (2000). Overexpression in *Escherichia coli*, purification, and characterization of *Sphingomonas* sp A1 alginate lyases. Protein. Express. Purif..

[35] Hirayama M, Hashimoto W, Murata K, Kawai S (2016). Comparative characterization of three bacterial exo-type alginate lyases. Int. J. Biol. Macromol..

[36] Sherman F (2002). Getting started with yeast. Methods Enzymol.

[37] Bradford MM (1976). A rapid and sensitive method for the quantitation of microgram quantities of protein utilizing the principle of protein-dye binding. Anal. Biochem..

[38] Czok, R. & Lamprecht, W. in Methods of Enzymatic Analysis, Vol. 3. (ed. H.U. Bergmeyer) 1446-1451 (Verlag Chemie GmbH, Weinheim, Germany; 1974).

[39] Kitagawa M (2005). Complete set of ORF clones of *Escherichia coli* ASKA library (a complete set of *E. coli* K-12 ORF archive):unique resources for biological research. DNA Res..

[40] Fukuda C, Kawai S, Murata K (2007). NADP(H) phosphatase activities of archaeal inositol monophosphatase and eubacterial 3′-phosphoadenosine 5′-phosphate phosphatase. Appl. Environ. Microbiol..

[41] Fujiwara H, Kawai S, Murata K (2013). Significance of sulfiredoxin/peroxiredoxin and mitochondrial respiratory chain in response to and protection from 100% O_2_ in *Saccharomyces cerevisiae*. Mitochondrion.

