# Supplementary material for: Crucial role of 4-deoxy-L-erythro-5-hexoseulose uronate reductase for alginate utilization revealed by adaptive evolution in engineered Saccharomyces cerevisiae
Source: Sci Rep. 2017 Jun 23;7:4206. doi: 10.1038/s41598-017-04481-3 (PMC5482797; doi:10.1038/s41598-017-04481-3)
Supplement: Supplementary file 1 — Supplementary information [file 41598_2017_4481_MOESM1_ESM.pdf]

Supplementary information for

Crucial role of 4-deoxy-L-*erythro*-5-hexoseulose uronate reductase for alginate utilization revealed by adaptive evolution in engineered *Saccharomyces cerevisiae*

Fumiya Matsuoka[†], Makoto Hirayama[†], Takayuki Kashihara[†], Hideki Tanaka[†], Wataru Hashimoto[†], Kousaku Murata[§], Shigeyuki Kawai[*,†]

[†] *Laboratory of Basic and Applied Molecular Biotechnology, Division of Food Science and Biotechnology, Graduate School of Agriculture, Kyoto University, Uji, Kyoto, 611-0011, Japan*

[§] *Faculty of Science and Engineering, Department of Life Science, Setsunan University, 17-8 Ikeda-Nakamachi, Neyagawa, Osaka, 572-8508, Japan*

[*] Corresponding author.
kawais@kais.kyoto-u.ac.jp

Supplementary methods

Plasmids. All plasmids used in this study are listed in Supplementary Table S1. Genomic PCR was conducted using *S. cerevisiae* BY4742 genomic DNA as a template with either KOD-Plus Neo (Toyobo, Japan) for cloning or KOD FX Neo (Toyobo) for verification. The amplified sequence was confirmed after cloning. *DSF1* and *HXT17* were amplified by genomic PCR with primers (7 and 8 for *DFS1*, 5 and 6 for *HXT17*) and inserted into FseI and SalI of pAT426, respectively, yielding pMK5503. pMK5503 was digested by XhoI/SacI, and the resultant XhoI/SacI fragment carrying *DSF1* and *HXT17* was inserted with In-fusion into EcoRV of pFA6a-kanMX6, a PCR fragment amplified with primers 1 and 2 (a template; pFA6a-kanMX6), resulting in pMK5547. The pseudogene sequence (*YIL174w/175w/176c*, 902-b, corresponding to the sequence of 8,794–9,696 nt of chromosome IX; SGD) was amplified with genomic PCR (primers 3 and 4) and inserted into HpaI of pMK5547 with disruption of the original HpaI, yielding pMK5550 in which only one HpaI exists at 500 nt in *YIL174w/175w/176c*.

pMK5076, which contained the *cyc8Δ1139–1164* allele from MK4416 (YCplac33-*cyc8* (4456) [1]), was digested with EcoRI/NaeI, blunted, and ligated, resulting in pMK5482 lacking *CEN4/ARS1*. Only one EcoNI exists in 1,822 nt in the *cyc8Δ1139–1164* allele.

We synthesized the genes, *yopt_kdgK*, *yopt_eda*, *yopt_A1-R'*, *yopt_DHT1*, and *yopt_A1-I* that correspond to *E. coli kdgK* [2], *E. coli eda* [3], *Sphingomonas* sp. A1 *A1-R'* [4], *A. cruciatus DHT1* [5], and *Sphingomonas* sp. A1 *A1-I* [6], with codons that are optimized for *S. cerevisiae* [7](Supplementary Figure S1). The *yopt_kdgK* and *yopt_eda*, each of which is flanked by 5'-*TEF1t* and 3'-*CYC1t*, were inserted into XhoI and SacI of pRS425, respectively, producing pMK4502. A possible pseudogene, *HXT12* (*YIL170w*), was amplified with genomic PCR with primers 9 and 10, and inserted into PstI of pMK4502 with disruption of this original PstI, producing pMK5461 in which only one PstI exists at 658 nt in *HXT12* (1,374 b). The pMK5461 was digested with Eco47III to remove only the 2 μ region, and then self-ligated, yielding pMK5471.

The *yopt_A1-R'*, which is flanked by 5'-*TEF1t* and 3'-*CYC1t*, was inserted into SalI of pRS423, producing pMK5090. The pMK5090 was digested with SnaBI/XcmI, blunted, and then self-ligated to remove only the 2 μ region, yielding pMK5090-1. A probable pseudogene, *YOL153C*, was amplified with genomic PCR with primers 11 and 12 and inserted into BamHI of pMK5090-1 with disruption of this original BamHI, producing pMK5474 in which only one NcoI exists at 903 nt in *YOL153C* (1,746 b). The *yopt_DHT1* was inserted into SalI of

pAT422, resulting in pMK5531. The *yopt_DHT1* flanked by 5'-*TDH3p* and 3'-*TDH3t* was amplified by PCR with primers 13 and 14 and with pMK5531 as a template, and was inserted into SacI of pMK5474 with a disruption of the original SacI, yielding pMK5552 in which only one NcoI exists at 903 nt in *YOL153C* (1,746 b). A c.50A>G mutation was introduced in *A1-R'* with inverse PCR using primers 42 and 43 and with pMK5552 as a template, yielding pMK5552-1. The *A1-R'*(c.50A>G), that was again amplified with PCR using primers 44 and 45 and with the pMK5552-1 as a template, was inserted into EcoRV/XhoI of pMK5552, creating pMK5859 that is the pMK5552 in which only c.50A>G was introduced into *A1-R'*.

The *yopt_A1-I* was amplified by PCR with primers 26 and 27, inserted into NdeI/BamHI of pET-14b (Novagen), yielding pMK5631. The c.50A>G was also introduced into *A1-R'* in pMK4699 (A1-R' in pET-21b) [4] with PCR using primers 46 and 47 and with pMK4699 as a template, resulting in pMK5922.

Strains. All strains used in this study are listed in Supplementary Table S2. Transformation of *S. cerevisiae* was conducted with the lithium acetate/single-stranded carrier DNA/polyethylene glycol method [8]. *ADE2* and *TRP1* were deleted from BY4742 as described previously using 5-fluoroorotic acid [9], yielding MK5315. In order to replace *CYC8* with the *cyc8Δ1139–1164* allele in the genomic DNA of the D452-2 strain, pMK5482 linearized by the digestion of EcoNI, was introduced into the D452-2 strain, resulting in MK5502, as described using 5-fluoroorotic acid [9].

To introduce *yopt_kdgK* and *yopt_eda*, pMK5471 linearized by the digestion of PstI was introduced into the D452-2 and MK5315 strains, producing MK5517 and MK5524, respectively, in which the targeting sites were confirmed by genomic PCR with primers (21 and 22; 18 and 23) followed by sequencing. To further introduce both *yopt_A1-R'* and *yopt_DHT1*, pMK5552 linearized by the digestion of NcoI was introduced into the MK5517 and MK5524 strains, yielding MK5590 and MK5591, respectively, in which the targeting sites were confirmed by genomic PCR with primers (21 and 24; 23 and 25). Likewise, to integrate both *yopt_A1-R'*(c.50A>G) and *yopt_DHT1*, pMK5859 was linearized by the digestion of NcoI and was again introduced into the MK5517 and MK5524 strains, yielding MK5906 (D_DEH+_E17G) and MK5909 (BY_DEH+_E17G), respectively. Again, the targeting sites were confirmed by genomic PCR with primers (21 and 24; 23 and 25).

To introduce *DSF1/HXT17*, pMK5550 was linearized by the digestion of HpaI and was introduced into the MK5590 and MK5591 strains, yielding MK5609 (D_DEH+) and MK5622 (BY_DEH+), and also into the BY4742 and D452-2 strains, yielding MK5580 and MK5583,

respectively. The targeting sites were confirmed by genomic PCR with primers (MK5580 and MK5609, 15 and 16; 17 and 18; MK5583, 19 and 16; 17 and 20). To determine the targeting site in MK5622, genomic DNA from MK5622 was digested by either NheI or XbaI, purified, self-ligated, and introduced into *Escherichia coli* DH5α. The plasmid was purified from the *E. coli* transformant showing kanamycin-resistance due to *KanMX* from pMK5550, and the sequence of the plasmid was determined. The ρ^0 mutants were created with 25 μg/mL ethidium bromide as described [10].

E. coli BL21(DE3)pLysS (Novagen) was transformed with pMK5631, resulting in the MK5632 strain for the expression of A1-I with an N-terminal His-tag. *E. coli* BL21(DE3) (Novagen) was transformed with either pMK4699 or pMK5922, yielding MK4700 or MK5923, respectively, for the preparations of rA1-R' and rA1-R'_E17G without any His-tag.

Measurement of oxygen consumption rate. Oxygen consumption was measured as described [11] with a slight modification. Briefly, yeast cells that were pre-grown on YPGA solid medium were inoculated into 1.0 mL SE liquid medium with a final A_{600} of 0.1 and were grown to reach an A_{600} of about 0.3. The cells were collected, resuspended in a fresh SE to an A_{600} of about 0.2. This suspension (150 μL) was mixed with 10 μL of MitoXpress (Luxcel Biosciences, Ireland), put in a well of a flat-bottom clear 96-well plate that was covered with mineral oil. The plate was monitored on an Infinite 200 Pro (Tecan) at 30˚C with a filter (360 nm extinction and 650 nm emission) every 1–2 min over 90 min by taking two intensity readings at delay times of 30 and 70 μs and a gate time of 30 μs. Based on the time-resolved fluorescence signal that was the average of 3 reactions, lifetime (μs) (τ) was calculated. From the τ profiles, the initial slopes (μs min^{-1}), reflecting oxygen consumption rates, were determined [11].

Determination of the concentrations of rA1-R' and rA1-R'_E17G. The concentrations of the purified A1-R' and E17G A1-R' were 0.676 and 0.326 mg/mL, respectively, based on the Bradford procedure, with bovine serum albumin as the standard. Kinetic parameters were calculated using these values. The concentrations of each of the purified enzymes were 0.477 and 0.303 mg/mL, respectively, when determined from the extinction coefficient (18,450) at A_{280} of both rA1-R' and rA1-R'_E17G (calculated molecular mass of both enzymes; 27,300), in which the protein solution (1 mg/mL) gives an A_{280} of 0.677.

A

ctcgag*catagcttcaaaatgtttctactccttttttactcttccagattttctcggactccgcgcatcgccgtaccacttcaaaacacccaagcacag catactaaatttcccctctttcttcctctagggtgtcgttaattacccgtactaaaggtttggaaaagaaaaaagagaccgcctcgtttctttttcttcgtc gaaaaaggcaataaaaatttttatcacgtttctttttcttgaaaattttttttttgatttttttctctttcgatgacctcccattgatatttaagttaataaacggt cttcaatttctcaagtttcagtttcatttttcttgttctattacaactttttttacttcttgctcattagaaagaaagcatagcaatctaatctaagtttt*cctagg actATGTCCAAGAAGATTGCCGTCATCGGTGAATGTATGATTGAATTGTCTGAAAAGGGTGCTGAT GTCAAGAGAGGTTTCGGTGGTGACACCTTGAACACCTCTGTTTACATCGCTAGACAAGTTGATCC AGCTGCTTTGACCGTCCACTACGTCACCGCTTTGGGTACTGACTCCTTCTCTCAACAAATGTTGG ATGCTTGGCACGGTGAAAACGTCGATACCTCTTTGACCCAAAGAATGGAAAACAGATTGCCAGGT TTGTACTACATTGAAACCGACTCTACTGGTGAAAGAACTTTCTACTACTGGAGAAACGAAGCCGCT GCCAAGTTCTGGTTGGAATCTGAACAATCTGCCGCTATCTGTGAAGAATTGGCTAACTTCGACTA CTTGTACTTGTCTGGTATCTCCTTGGCTATCTTGTCTCCAACTTCCAGAGAAAAGTTGTTGTCCTT GTTGAGAGAATGTAGAGCTAACGGTGGTAAGGTTATCTTCGACAACAACTACAGACCAAGATTGT GGGCTTCCAAGGAAGAAACTCAACAAGTTTACCAACAAATGTTGGAATGTACTGACATCGCCTTC TTGACCTTGGACGACGAAGATGCTTTGTGGGGTCAACAACCAGTTGAAGATGTCATCGCTAGAAC CCACAACGCTGGTGTCAAGGAAGTTGTCGTCAAGAGAGGTGCTGACTCTTGTTTGGTCTCTATCG CTGGTGAAGGTTTGGTTGACGTTCCAGCTGTTAAGTTGCCAAAGGAAAAGGTTATTGACACCACT GCCGCTGGTGACTCCTTCTCTGCTGGTTACTTGGCTGTCAGATTGACTGGTGGTTCTGCTGAAAA CGCTGCCAAGAGAGGTCACTTGACTGCTTCTACTGTTATTCAATACAGAGGTGCTATCATTCCAA GAGAAGCTATGCCAGCTTAGacaggcgcgccttcctttg***tcgagtcatgtaattagttatgtcacgcttacattcacgccctcc ccccacatccgctctaaccgaaaaggaaggagttagacaacctgaagtctaggtccctatttatttttttatagttatgttagtattaaga acgttatttatatttcaaatttttcttttttttctgtacagacgcgtgtacgcatgtaacattatactgaaaaccttgcttgagaaggttttggg acgctcgaaggctttaatttgcggcc***ctcgag

B

gagct*catagcttcaaaatgtttctactccttttttactcttccagattttctcggactccgcgcatcgccgtaccacttcaaaacacccaagcacagc atactaaatttcccctctttcttcctctagggtgtcgttaattacccgtactaaaggtttggaaaagaaaaaagagaccgcctcgtttctttttcttcgtc gaaaaaggcaataaaaatttttatcacgtttctttttcttgaaaattttttttttgatttttttctctttcgatgacctcccattgatatttaagttaataaacggt cttcaatttctcaagtttcagtttcatttttcttgttctattacaactttttttacttcttgctcattagaaagaaagcatagcaatctaatctaagtttt*cggacc gtctcatATGAAGAACTGGAAGACCTCTGCTGAATCCATCTTGACCACTGGTCCAGTTGTTCCAGTCA TCGTCGTTAAGAAGTTGGAACACGCTGTTCCAATGGCTAAGGCTTTGGTTGCTGGTGGTGTTAGA GTCTTGGAAGTCACCTTGAGAACTGAATGTGCCGTTGATGCCATCAGAGCTATTGCTAAGGAAGT TCCAGAAGCCATTGTTGGTGCTGGTACTGTCTTGAACCCACAACAATTGGCTGAAGTCACCGAAG CTGGTGCTCAATTCGCCATCTCTCCAGGTTTGACCGAACCATTGTTGAAGGCCGCTACTGAAGGT ACTATTCCATTGATTCCAGGTATCTCCACTGTCTCTGAATTGATGTTGGGTATGGACTACGGTTTG AAGGAATTCAAGTTCTTCCCAGCTGAAGCTAACGGTGGTGTCAAGGCTTTGCAAGCCATTGCTGG TCCATTCTCTCAAGTCAGATTCTGTCCAACTGGTGGTATCTCTCCAGCTAACTACAGAGACTACTT GGCTTTGAAGTCTGTCTTGTGTATCGGTGGTTCCTGGTTGGTTCCAGCTGACGCTTTGGAAGCTG GTGACTACGATAGAATCACCAAGTTGGCTAGAGAAGCTGTTGAAGGTGCTAAGTTGTAAacagctag cttttcctttg***tcgagtcatgtaattagttatgtcacgcttacattcacgccctccccccacatccgctctaaccgaaaaggaaggagtta gacaacctgaagtctaggtccctatttatttttttatagttatgttagtattaagaacgttatttatatttcaaatttttcttttttttctgtacagac gcgtgtacgcatgtaacattatactgaaaaccttgcttgagaaggttttgggacgctcgaaggctttaatttgcggcc***gagctc

C

gtcgac*catagcttcaaaatgtttctactccttttttactcttccagattttctcggactccgcgcatcgccgtaccacttcaaaacacccaagcacag catactaaatttcccctctttcttcctctagggtgtcgttaattacccgtactaaaggtttggaaaagaaaaaagagaccgcctcgtttctttttcttcgtc gaaaaaggcaataaaaatttttatcacgtttctttttcttgaaaattttttttttgatttttttctctttcgatgacctcccattgatatttaagttaataaacggt cttcaatttctcaagtttcagtttcatttttcttgttctattacaactttttttacttcttgctcattagaaagaaagcatagcaatctaatctaagtttt*cgtacg aaaATGTTCTCTGACTTGAAGGGTAAGAGAATCTTGATCACTGGTTCTACCG**A**AGGTATCGGTATG GCTACTGCTATCGAATTGGCTAGATACGGTGCTGTTGTTGGTTTGAACTCTCACGTTGACCCAGC TGACCCAGCCTTGTTGTTGGGTAAGTTGAGAGAAGCTGGTGGTGATGGTGCCTTCTTCAGAGCT GACATCACCAAGACCGCTGAATGTCAAAGATTGGTTTCTGCTTTCGTTGAAAGATTCGACGGTATT GATGTCTTGATCAACAACGCTGGTGGTTTGGCTGGTAGATCTAACTTGGAAAACATTGATGACGC TTTCTACGACAGAGTCATGGACTTGAACGGTAGATCTGTCTTGATGATGACCAAGTTCGCTATCC CACACTTGAGAGCTTCTGCTAAGGCTTCTGGTACTACCTCTGCTGTCATTTCCACTGGTTCCATCG CTGCCAGAGAAGGTGGTGGTATTGGTGCTGGTGTCTACGCTGCCTCCAAGGCTTGGTTGCACGA CATTCACAGAAACTGGGTCAAGGAATTCACCAAGGATTCCATCAGATTCAACATTGTTGCTCCAG GTACTGTTGATACTGCTTTCCACGCTGACAAGTCTGACGAATTGAAGACCAGAATCGCCAACTCC ATCCCAATGGGTAGATTCGGTACTGTTCAAGAATTGGCTCCAGCCTACGTCTTCTTCGCTTCTCAC

GCTGCCTCTGGTTACATCACTGGTCAAATCTTGGATGTCAACGGTGGTCAAATCTGTCCATAGaca
ggccggccttcctttg***tcgagtcatgtaattagttatgtcacgcttacattcacgccctccccccacatccgctctaaccgaaaaggaag***
gagttagacaacctgaagtctaggtccctatttattttttatagttatgttagtattaagaacgttatttatatttcaaatttttctttttttctgta
cagacgcgtgtacgcatgtaacattatactgaaaaccttgcttgagaaggttttgggacgctcgaaggctttaatttgcggccgtcgac

D

AACAAACAAA*GTCGAC*ATGGGTATCTTGGACAAGTTGATCAAGAACGAATCTATGAAGTCTGACC
CAAAGGAAATCTACGGTTGGAGAATCTGGGCTTTGGCTTCTTCTGCTTGTTTCGGTGGTATGTTG
TTCGGTTGGGACATCGGTGCTATCGGTGGTATCTTGGTTATGCCATCTTTCCAAGAAAAGTTCGG
TTTGGCTGAAAAGTCTGAATCTGAATTGGCTGACGTTGAATCTAACATCGTTTCTGTTTTGCAAGC
TGGTTGTTTCGCTGGTTCTTTGATCGCTTACTGGATCGCTGACAGATGGGGTAGAAAGCCATCTT
TGTTGGCTTCTGCTGCTATGTCTACTATCGGTGTTGTTATCCAAACTGCTTCTTCTGGTCACTTGG
CTGCTTTGTTCGTTGGTAGATTCTTGGCTGGTTTGGGTGTTGGTGCTGCTTCTATGTTGACTCCAT
TGTACGTTTCTGAAAACGCTCCAAGATCTATCAGAGGTGCTTTGACTGGTTTGTACCAATTGAACA
TCACTATCGGTATCATGTTGTCTTTCTGGGTTAACTTCGGTTCTTTGAGACACTCTGAAGGTGACA
TCCAATGGGAAGTTCCATTGGCTACTCAAATGTTGGCTGCTGTTTTCATGTTCGTTGGTATCTCTT
TGTGTGGTGAATCTCCAAGATTCTTGGCTAAGCAAGACAACTGGGAAGCTGCTTCTGCTGTTTTG
TCTAAGTTGAGAAACTTGCCAGCTGACCACACTTACATCGCTCAAGAATTGCAAGACATGGCTGA
CCAATTGGAAAAGGAAAGAGGTCACTCTGACAACAACTCTTTCTGGGGTTTGCACAGAGACATGT
GGACTGTTCCAACTAACAGAAAGAGAGCTTTGATCTCTATCGGTTTGATGATCTGTCAACAAATGA
CTGGTGTTAACGCTATCAACTACTACGCTCCAAAGATCTTCAACGGTTTGGGTATCCAAGGTCCAT
CTAACGGTTTGTTCGCTACTGGTGTTTACGGTATCGTTAAGGTTGTTGGTTGTGCTTTGTTCGTTT
TGTTCGCTGCTGACTCTGTTGGTAGAAGATTGTCTTTGTTGTGGACTGCTATCGCTCAAGGTATCT
TCATGTTCATCATCGGTTGTTACGTTTTGACTAACCCACCAGTTGAAGGTGCTCCAATCCCAGCTT
TCGGTTACGTTGCTTTGGTTTCTATCTTCTTGTTCGTTTTGTGTTTCGAAGTTGGTTGGGGTCCAG
CTTGTTGGATCTTGGTTTCTGAAATCCCACAAGCTAGATTGAGAGCTTTGAACGTTGCTTTGGCTG
CTGCTACTCAATGGTTGTTCAACTTCGTTGTTGCTCAAGCTGTTCCACACATGTTGATCACTACTG
GTGAAGGTGGTTACGGTACTTACTTCATCTTCGGTTCTTTCTCTTTCTGTATGTTCTTCTTCACTTG
GTTCTTGATCCCAGAAACTAAGGGTGTTTCTTTGGAAAAGATGGACGCTTTGTTCGGTGTTAAGG
CTCCATTGGGTGGTGAAGAAGGTAACCCAGAATTCTTGGAAAAGTCTGGTCCACACACTGACGCT
GACGCTGGTTCTGGTAAGTTCGCTGAAACTACTCACATCGAAAGAAAGTGA*GTCGAC*ACGCGTG
CGG

E

ATGCACCCATTCGATCAAGCTGTCGTCAAGGACCCAACTGCTTCTTACGTTGATGTCAAGGCTAG
AAGAACCTTCTTGCAATCTGGTCAATTGGATGACAGATTGAAGGCCGCTTTGCCAAAGGAATACG
ACTGTACCACTGAAGCTACTCCAAACCCACAACAAGGTGAAATGGTTATCCCAAGAAGATACTTG
TCTGGTAACCACGGTCCAGTCAACCCAGACTACGAACCAGTCGTTACCTTGTACAGAGACTTCGA
AAAGATCTCTGCTACCTTGGGTAACTTGTACGTTGCTACTGGTAAGCCAGTTTACGCTACTTGTTT
GTTGAACATGTTGGACAAGTGGGCCAAGGCTGATGCTTTGTTGAACTACGATCCAAAGTCTCAAT
CTTGGTACCAAGTTGAATGGTCTGCCGCTACTGCCGCCTTCGCTTTGTCCACCATGATGGCTGAA
CCAAACGTTGACACTGCTCAAAGAGAAAGAGTCGTTAAGTGGTTGAACAGAGTTGCTAGACACCA
AACCTCCTTCCCAGGTGGTGACACCTCCTGTTGTAACAACCACTCCTACTGGAGAGGTCAAGAAG
CTACCATTATCGGTGTCATTTCCAAGGATGACGAATTGTTCAGATGGGGTTTGGGTAGATACGTT
CAAGCTATGGGTTTGATCAACGAAGATGGTTCTTTCGTTCACGAAATGACCAGACACGAACAATC
CTTGCACTACCAAAACTACGCTATGTTGCCATTGACCATGATCGCTGAAACTGCTTCCAGACAAG
GTATCGACTTGTACGCTTACAAGGAAAACGGTAGAGACATTCACTCTGCTAGAAAGTTCGTCTTC
GCCGCTGTCAAGAACCCAGACTTGATCAAGAAGTACGCTTCTGAACCACAAGATACTAGAGCTTT
CAAGCCAGGTAGAGGTGACTTGAACTGGATTGAATACCAAAGAGCTAGATTCGGTTTCGCTGACG
AATTGGGTTTCATGACCGTTCCAATCTTCGATCCAAGAACTGGTGGTTCTGGTACTTTGTTGGCTT
ACAAGCCACAAGGTGCTGCCGCTCAAGCTCCAGTTTCTGCTCCAGCTGCCGCTCACTCCTCTATC
GACTTGTCTAAGTGGAAGTTGCAAATTCCAGTTGACCCAATCGACGTTGCCACCAGAGACTTGTT
GAAGGGTTACCAAGACAAGTACTTCTACGTTGACAAGGATGGTTCCTTGGCTTTCTGGTGTCCAG
CTTCTGGTTTCAAGACCACTGCTAACACCAAGTACCCAAGATCTGAATTGAGAGAAATGTTGGAC
CCAGACAACCACGCTGTCAACTGGGGTTGGCAAGGTACTCACGAAATGAACTTGAGAGGTGCTG
TCATGCACGTTTCTCCATCTGGTAAGACCATCGTCATGCAAATTCACGCTGTCATGCCAGACGGT
TCTAACGCTCCACCATTGGTCAAGGGTCAATTCTACAAGAACACCTTGGACTTCTTGGTCAAGAA
CTCTGCCGCTGGTGGTAAGGACACTCACTACGTCTTCGAAGGTATTGAATTGGGTAAGCCATACG
ACGCTCAAATCAAGGTCGTTGATGGTGTCTTGTCTATGACTGTCAACGGTCAAACCAAGACTGTT
GACTTCGTTGCCAAGGACGCTGGTTGGAAGGACTTGAAGTTCTACTTCAAGGCTGGTAACTACTT

GCAAGACAGACAAGCTGATGGTTCTGACACTTCTGCCTTGGTCAAGTTGTACAAGTTGGATGTTA
AGCACTCCTCTTGA

Supplementary Fig. S1. Sequences of synthesized *yopt_kdgK* (A), *yopt_eda* (B), *yopt_A1-R'* (C), *yopt_DHT1* (D), and *yopt_A1-I* (E), with codons optimized for *S. cerevisiae*.

A: XhoI sites [ctcgag], underlined 1-6, 1626–1631 (6 bp), *TEFp* 7–410 (404 bp) italic, linker AvrII [cctagg], PflFI [gactatgtc] 411–419 (9 bp), *E. coli kdgK* 420–1349 (930 bp) capital letter, linker AscI [ggcgcgcc] 1350–1368 (19 bp), *CYC1t* 1369–1625 (257 bp) bold and italic.

B: SacI site [gagctc] 1–6 (6 bp), *TEFp* 6–409 (404 bp), linker RsrII [cggaccg], BsmBI [cgtctca] 410–422 (13 bp), *E. coli eda* 423–1064 (642 bp), linker BmtI [gctagc] 1065–1083 (19 bp), *CYCt* 1084–1340 (257 bp), BamHI site [ggatcc] 1341–1346 (6 bp).

C: SalI sites [gtcgac] 1–6, 1470–1475 (6 bp), *TEFp* 7–410 (404 bp), linker BsiWI [cgtacg] 411–419 (9 bp), *Sphingomonas* sp. A1 *A1-R'* 420–1193 (774 bp), linker FseI [ggccggcc] 1194–1212 (19 bp), *CYCt* 1213–1469 (257 bp). The 50A, at which the causal mutation occurred, is in bold and double-underlined.

D, E: Sequences needed for in-fusion are underlined.

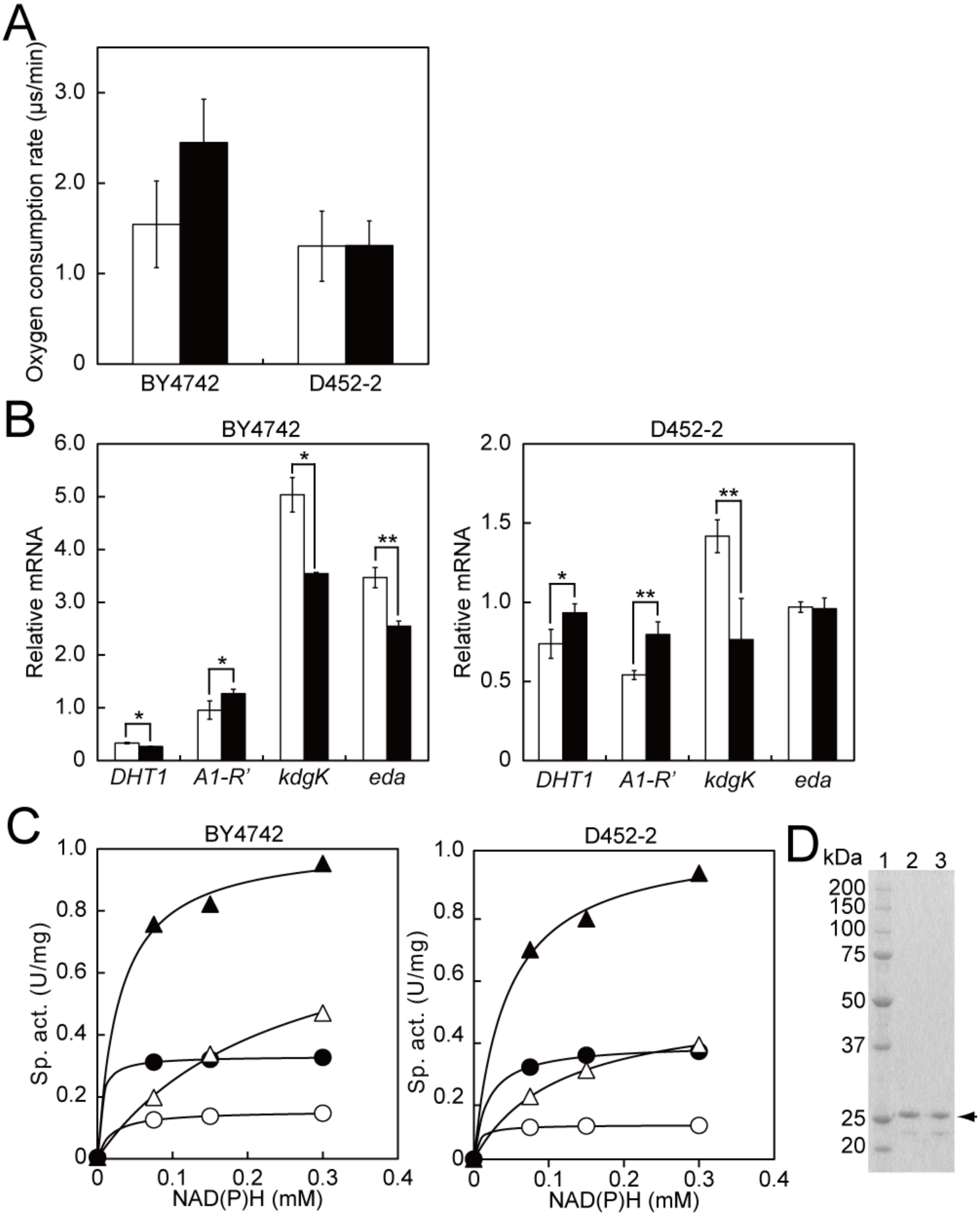

Supplementary Fig. S2. The c.50A>G in *yopt_A1-R'* was the causal mutation for enhanced DEH reductase activity and growth in DEH medium. A: Oxygen consumption rates of DEH+ (white bar) and DEH++ (black bar) on BY4742 and D452-2 backgrounds grown in SE medium were measured. Average and SD are shown (n=3).

B: Quantitative PCR. The transcripts for each gene were isolated from both the DEH+ (white bar) and DEH++ (black bar) strains on BY4742 and D452-2 backgrounds grown in DEH (HC-LN) liquid medium, quantified, and normalized against those for *ACT1* as described in Methods. *; $p<0.05$, **; $p<0.01$. C: NADH (circles)- and NADPH (triangles)-dependent DEH reductase activities in the cell extracts from DEH+ (open symbols) and DEH++ (closed symbols) strains on BY4742 and D452-2 backgrounds. D: SDS-PAGE of the purified rA1-R' (lane 2, 0.5 μg) and rA1-R'_E17G (lane 2, 0.5 μg). Precision Plus Protein standards (Bio-Rad) were used as markers (lane 1). An arrow indicates the purified rA1-R' and rA1-R'_E17G.

Supplementary Table S1. Plasmids used in this study

Plasmid	Description	Source
pAT426	Ap^r, 2 μm, *URA3*, *TDH3p-TDH3t*, *ADH1p-ADH1t*	[12]
pAT422	Ap^r, 2 μm, *ADE2*, *TDH3p-TDH3t*, *ADH1p-ADH1t*	[12]
YCplac33	Ap^r, *CEN*, *URA3*	[13]
pFA6a-kanMX6	Ap^r, Km^{r} [a]	[14]
pRS423	Ap^r, 2 μm, *HIS3*	[15]
pRS425	Ap^r, 2 μm, *LEU2*	[15]
pMK5503	*HXT17* in SalI and *DSF1* in FseI of pAT426; *ADH1p-HXT17-ADH1t*/*TDH3p-DSF1-TDH3t*	This study
pMK5547	*TDH3p-DSF1-TDH3t*/*ADH1p-HXT17-ADH1t* in EcoRV of pFA6a-kanMX6	This study
pMK5550	*YIL174w/175w/176c* in HpaI of pMK5547	This study
pMK5076 =YCplac33-cyc8 (4416)	5'-2,337-b (-1 to -2,337)-*cyc8Δ1139–1164* (*cyc8* allele from MK4416, 2,901-b)-3'-1,389-b (+1 to +1,339) in SmaI of YCplac33	[1]
pMK5482	pMK5076 lacking *CEN4/ARS1*	This study
pMK4502	*TEF1p-yopt_kdgK-CYC1t* in XhoI and *TEF1p-yopt_eda-CYC1t* in SacI of pRS425	This study
pMK5461	*HXT12* in PstI of pMK4502	This study
pMK5471	pMK5461 (Δ2 μm)	
pMK5090	*TEF1p-yopt_A1-R'-CYC1t* in SalI of pRS423	This study
pMK5090-1	pMK5090 (Δ2 μm)	
pMK5474	*YOL153C* in BamHI of pMK5090-1	This study
pMK5531	*yopt_DHT1* in SalI of pAT422	This study
pMK5552	*TDH3p-yopt_DHT1-TDH3t* in SacI of pMK5474	This study
pET-14b	Ap^r, Expression plasmid for *E. coli*	Novagen
pMK5631	*yopt_A1-I* in NdeI/BamHI of pET-14b	This study
pMK5859	pMK5552 in which *A1-R'* was replaced with *A1-R'*(c.50A>G)	This study
pMK4699	*A1-R'* in NdeI/BamHI of pET-21b	[4]
pMK5922	*A1-R'*(c.50A>G) in NdeI/BamHI of pET-21b	This study

Supplementary Table S2. Strains used in this study

Strain	Description	Source
S. cerevisiae		
BY4742	MATα *his3Δ1 leu2Δ0 lys2Δ0 ura3Δ0*	Euroscarf
D452-2	MATα *leu2 his3 ura3 can1*	Dr. T. Kodaki, Kyoto Univ. [16]
MK4416	BY4742 *cyc8Δ1139-1164* [a]	[1]
MK5315	BY4742 *ade2Δ0 trp1Δ63*	This study
MK5502	D452-2 *cyc8Δ1139-1164* [a]	[1]
MK5580	BY4742 *YJL222w-A-kanMX- TDH3p-DSF1-TDH3t-ADH1p-HXT17-ADH1t- YJL222w-A*	This study
MK5583	D452-2 *YCR102w-A-kanMX- TDH3p-DSF-TDH3t-ADH1p-HXT17-ADH1t-YCR102w-A*	This study
MK5517	D452-2 *YJL219w::TEF1p-yopt_eda-CYC1t-LEU2-TEF1p-yopt_kdgK-CYC1t-YJL219w*	This study
MK5524	MK5315 *YJL219w-TEF1p-yopt_eda-CYC1t-LEU2-TEF1p-yopt_kdgK-CYC1t-YJL219w*	This study
MK5590	MK5517 *YOL153C-TDH3p-yopt_DHT1-TDH3t-HIS3-TEF1p-yopt_A1-R'-CYC1t-YOL153C*	This study
MK5591	MK5524 *YOL153C-TDH3p-yopt_DHT1-TDH3t-HIS3-TEF1p-yopt_A1-R'-CYC1t-YOL153C*	This study
MK5609	MK5590 *YJL222w-A-kanMX-TDH3p-DSF1-TDH3t-ADH1p-HXT1-ADH1t-YJL222w-A* = D_DEH+	This study
MK5622	MK5591 *kanMX-TDH3p-DSF1-TDH3t-ADH1p-HXT17-ADH1t* =BY_DEH+	This study
MK5717	Evolved MK5609 = D_DEH++	This study
MK5719	Evolved MK5622 = BY_DEH++	This study
MK5906	MK5517 *YOL153C-TDH3p-yopt_DHT1-TDH3t-HIS3-TEF1p-yopt_A1-R'* (c.50A>G)-*CYC1t-YOL153C* = D_DEH+_E17G	This study
MK5909	MK5524	This study

	YOL153C-TDH3p-yopt_DHT1-TDH3t-HIS3-TEF1p-yopt_ A1-R'(c.50A>G)-*CYC1t-YOL153C* = BY_DEH+_E17G	
E. coli		
DH5α	F^- φ80*dlacZ*ΔM15 Δ(*lacZYA-argF*)U169 *deoR recA1* *endA*1 *hsdR*17 (r_k^-, m_k^+) *phoA supE*44 λ^- *thi*-1 *gyrA*96 *relA*1	Toyobo
BL21(DE3) pLysS	Host for pET-syetem	Novagen
BL21(DE3)	Host for pET-syetem	Novagen
MK5632	BL21(DE3)pLysS pMK5631; for expression of A1-I	This study
MK4700	BL21(DE3) pMK4699; for expression of A1-R'	[4]
MK5923	BL21(DE3) pMK5922; for expression of A1-R'_E17G	This study
MK5805	ASKA(-) JW1839; for expression of Eda	NBRP [17]

[a] c.1139_1164del26 (p.Gln380AlafsX9) [1].

Supplementary Table S3. Primers used in this study

No.	Primer	Sequence	Description
1	pAT_dSacI_15b_ pFA6a_EV_Fw	CAGGATTTAATTAAG ATCAGATCCACTAGT GGCC	Amplification of pFA6a-kanMX6, underlined sequence corresponds to 15 b around SacI of pMK5503
2	pAT_XhoI_16b_p FA6a_EV_Rv	CAGAGGACAACTCGA GATCATCGATGAATT CGAGCTC	Amplification of pFA6a-kanMX6, underlined sequence corresponds to 165 b around XhoI of pMK5503
3	pFA6a_ dHpaI_15b_ YIL174-176_Fw	TCGATAAGCCAGGTT TAGTTTGCGATAGTG TAGATAC	Amplification of *IX8794*, underlined sequence corresponds to HpaI of pFA6a-kanMX6
4	pFA6a_ dHpaI_15b_ YIL174-176_Rv	TCATTAATGCAGGTT CTAACAGTATACGAC TCCCAAC	Amplification of *IX8794*, underlined sequence corresponds to HpaI of pFA6a-kanMX6
5	pAT_SalI_16b_H XT17_1_Fw	AACAAACAAAGTCGA CATGCAATCATCCAC TGAAAGTG	Amplification of *HXT17*, underlined sequence corresponds to SalI of pAT426
6	pAT_SalI_16b_H XT17_1695Rv	CCGCACGCGTGTCGA CTCAATCAGAACCCT TTGAGAAC	Amplification of *HXT17*, underlined sequence corresponds to SalI of pAT426
7	pAT_FseId15b_D SF1_Fw	CTAGGGTTTAAAC*GG* ATGACAAAATCAGAC GAAAC	Amplification of *DSF1*, underlined sequence partially corresponds to FseI of pAT426
8	pAT_FseId15b_D SF1_Rv	ATAAGAAATTCGCGG TCACACTTGGTCTAA AATTTC	Amplification of *DSF1*, underlined sequence partially corresponds to FseI of pAT426
9	pRS425_dPstI_H XT12_Fw	TTGATATCGAATTCC ATGGGTTTGATTGTCT CAATATTC	Amplification of *HXT12*, underlined nucleotide partially corresponds to PstI of pRS425
10	pRS425_dPstI_H XT12_Rv	GTGGATCCCCCGGGC TCAGCTGGAAAAGAA CCTCTTG	Amplification of *HXT12*, underlined nucleotide partially corresponds to PstI of pRS425
11	pRS423_dBamHI _YOL153C_Fw	GCAGCCCGGGGGATC ATGACAGAGACTCAT	Amplification of *YOL153C*, underlined sequence partially

		CACGC	corresponds to BamHI of pRS423
12	pRS423_dBamHI_YOL153C_Rv	TAGAACTAGTGGATCTTAATCATGGCCATACTCGTTG	Amplification of *YOL153C*, underlined sequence partially corresponds to BamHI of pRS423
13	pRS_dSacI_TDHp_Fw	CCGCCACCGCGGTGGCCATATTGTACACCCCCGCG	Amplification of *TDH3p-yopt_DHT1-TDH3t*, to insert into SacI of pMK5474. Underlined nucleotide partially corresponds to SacI of pMK5474.
14	pRS_dSacI_TDHt_Rv	GGGAACAAAAGCTGGCGCCAAGCGCGCAATTAACC	Amplification of *TDH3p-yopt_DHT1-TDH3t*, to insert into SacI of pMK5474. Underlined nucleotide partially corresponds to SacI of pMK5474.
15	YIL176c_+163_+144_Rv	CGTCATTGATCAAATAGGTC	Confirming the targeting site of *DSF1/HXT17*
16	pFA6a-kanMX4_1618-1637_Rv	GGCCGATTCATTAATGCAGG	Confirming g the targeting site of *DSF1/HXT17*
17	HXT17_596-617_Fw	CATTGTACCAACTTAACATGAC	Confirming the targeting site of *DSF1/HXT17*
18	HXT9_+139_+159Rv	CACCCTGTCAACTCGTGTAGC	Confirming the targeting sites of *DSF1/HXT17* and *yopt_kdgK*
19	ADH7_95_115_Rv	CAACATCAACGTCATGATCG	Confirming the targeting site of *DSF1/HXT17*
20	YCR102c_3_22_Rv	CTTCAATGACGACAGCCTTC	Confirming the targeting site of *DSF1/HXT17*
21	M13RV	GGAAACAGCTATGACCATG	Universal primer. Confirming the targeting sites of *yopt_kdgK/yopt_eda* and *yopt_DHT1/yopt_A1-R'*
22	HXT12_-16_-7_Fw	TTCATCTTTGGTTGGGATAC	Confirming the targeting site of *yopt_kdgK/yopt_eda*
23	M13FW	GTAAAACGACGGCCAGT	Universal primer. Confirming the targeting sites of *yopt_kdgK/yopt_eda* and *yopt_DHT1/yopt_A1-R'*
24	YOL153C_1688_1708Fw	CAGCAGTAGCATTTGTCTATG	Confirming the targeting site of *yopt_DHT1/yopt_A1-R'*

25	YOL153C_+310_+329Rv	CACACATTTCGGTACGAACA	Confirming the targeting site of *yopt_DHT1/yopt_A1-R'*
26	yA1-I_1_20_pET-14b_Fw	CGCGCGGCAGCCATATGCACCCATTCGATCAAGC	Amplification of *yopt_A1-I*, to insert into NdeI/BamHI of pET-14b. Underlined sequence corresponds to NdeI.
27	yA1-I_758_777_BamHI_16b_Rv	GTTAGCAGCCGGATCCTCAAGAGGAGTGCTTAACATC	Amplification of *yopt_A1-I*, to insert into NdeI/BamHI of pET-14b. Underlined sequence corresponds to BamHI.
28	P_{TDH3p}	CTTAAACTTCTTAAATTCTAC	Sequencing
29	P_{ADH1p}	CCTCGTCATTGTTCTCGTTC	Sequencing
30	T_{TDH3p}	CAAAGCTTTCAATCAATGAATC	Sequencing
31	T_{ADH1p}	GAAAGAGTTACTCAAGAATAAG	Sequencing
32	ACT1_848_871_F	TGGCCGGTAGAGATTTGACTGACT	qPCR for *ACT1*
33	ACT1_980_957_R	TCGAAGTCCAAGGCGACGTAACAT	qPCR for *ACT1*
34	Ac_DHT1_912_935_F	CGCTATCAACTACTACGCTCCAAA	qPCR for *yopt_DHT1*
35	Ac_DHT1_1030_1010_R	CGAACAAAGCACAACCAACAA	qPCR for *yopt_DHT1*
36	sph1210_373_393_F	ACCAAGTTCGCTATCCCACAC	qPCR for *yopt_A1-R'*
37	sph1210_495_476_R	AGCGTAGACACCAGCACCAA	qPCR for *yopt_A1-R'*
38	kdgK_578_596_F	TCGCCTTCTTGACCTTGGA	qPCR for *yopt_kdgK*
39	kdgK_721_700_R	CACCAGCGATAGAGACCAAACA	qPCR for *yopt_kdgK*
40	kdgA_528_551_F	GTCTGTCTTGTGTATCGGTGGTTC	qPCR for *yopt_edaK*

41	kdgA_640_616_R	ACAACTTAGCACCTTCAACAGCTTC	qPCR for *yopt_eda*
42	sph1210_33_65_mut_Fw	GATCACTGGTTCTACCGGAGGTATCGGTATGGC	Introduction of c.50A>G in *A1-R'* in pMK5552
43	sph1210_33_65_mut_Rv	GCCATACCGATACCTCCGGTAGAACCAGTGATC	Introduction of c.50A>G in *A1-R'* in pMK5552
44	pRS_XhoI_sph1210_Fw	CGGGCCCCCCCTCGAGGGTC	Amplification of *A1-R'*(c.50A.G)
45	pRS_EcoRV_sph1210_Rv	CTGCAGGAATTCGATATCAAG	Amplification of *A1-R'*(c.50A.G)
46	sph1210_A50Gmut_Fw	CACCGGCTCGACCGGGGGTATCGGGATGGC	Introduction of c.50A>G in *A1-R'* in pMK4699
47	sph1210_A50Gmut_Rv	GCCATCCCGATACCCCCGGTCGAGCCGGTG	Introduction of c.50A>G in *A1-R'* in pMK4699
48	TEF_pro_Fw	CAATTTCTCAAGTTTCAGTTTC	Sequencing
49	CYC_ter_Rv	CTTCAGGTTGTCTAACTCCTTC	Sequencing

References

1. Chujo, M., Yoshida, S., Ota, A., Murata, K. & Kawai, S. Acquisition of the ability To assimilate mannitol by *Saccharomyces cerevisiae* through dysfunction of the general corepressor Tup1-Cyc8. *Appl. Environ. Microbiol.* **81,** 9-16 (2015).
2. Cynkin, M. A. & Ashwell, G. Uronic acid metabolism in bacteria .4. Purification and properties of 2-keto-3-deoxy-D-gluconokinase in *Escherichia coli*. *J. Biol. Chem.* **235,** 1576-1579 (1960).
3. Egan, S. E. *et al.* Molecular characterization of the Entner-Doudoroff pathway in *Escherichia coli*: sequence analysis and localization of promoters for the *edd-eda* operon. *J. Bacteriol.* **174,** 4638-4646 (1992).
4. Takase, R., Mikami, B., Kawai, S., Murata, K. & Hashimoto, W. Structure-based conversion of the coenzyme requirement of a short-chain dehydrogenase/reductase involved in bacterial alginate metabolism. *J. Biol. Chem.* **289,** 33198-33214 (2014).
5. Enquist-Newman, M. *et al.* Efficient ethanol production from brown macroalgae sugars by a synthetic yeast platform. *Nature* **505,** 239-243 (2014).
6. Yoon, H. J. *et al.* Overexpression in *Escherichia coli*, purification, and characterization of *Sphingomonas* sp A1 alginate lyases. *Protein. Express. Purif.* **19,** 84-90 (2000).
7. Bennetzen, J. L. & Hall, B. D. Codon selection in yeast. *J. Biol. Chem.* **257,** 3026-3031 (1982).
8. Gietz, R. D. & Woods, R. A. Transformation of yeast by lithium acetate/single-stranded carrier DNA/polyethylene glycol method. *Methods Enzymol.* **350,** 87-96 (2002).
9. Yoshida, S., Tanaka, H., Hirayama, M., Murata, K. & Kawai, S. Production of pyruvate from mannitol by mannitol-assimilating pyruvate decarboxylase-negative *Saccharomyces cerevisiae*. *Bioengineered* **6,** 347-350 (2015).
10. Fox, T. D. *et al.* Analysis and manipulation of yeast mitochondrial genes. *Methods Enzymol.* **194,** 149-165 (1991).
11. Troppens, D. M., Dmitriev, R. I., Papkovsky, D. B., O'Gara, F. & Morrissey, J. P. Genome-wide investigation of cellular targets and mode of action of the antifungal bacterial metabolite 2,4-diacetylphloroglucinol in *Saccharomyces cerevisiae*. *FEMS Yeast Res.* **13,** 322-334 (2013).

12. Ishii, J. *et al.* Three gene expression vector sets for concurrently expressing multiple genes in *Saccharomyces cerevisiae*. *FEMS Yeast Res.* **14,** 399-411 (2014).
13. Gietz, R. D. & Sugino, A. New yeast-*Escherichia coli* shuttle vectors constructed with *in vitro* mutagenized yeast genes lacking six-base pair restriction sites. *Gene* **74,** 527-534 (1988).
14. Bähler, J. *et al.* Heterologous modules for efficient and versatile PCR-based gene targeting in *Schizosaccharomyces pombe*. *Yeast***,** 943-951 (1998).
15. Christianson, T. W., Sikorski, R. S., Dante, M., Shero, J. H. & Hieter, P. Multifunctional yeast high-copy-number shuttle vectors. *Gene* **110,** 119-122 (1992).
16. Hosaka, K., Nikawa, J., Kodaki, T. & Yamashita, S. A dominant mutation that alters the regulation of INO1 expression in *Saccharomyces cerevisiae*. *J. Biochem. (Tokyo).* **111,** 352-358 (1992).
17. Kitagawa, M. *et al.* Complete set of ORF clones of *Escherichia coli* ASKA library (a complete set of *E. coli* K-12 ORF archive):unique resources for biological research. *DNA Res.* **12,** 291-299 (2005).
